# Isotopic analyses suggest mammoth and plant in the diet of the oldest anatomically modern humans from far southeast Europe

**DOI:** 10.1038/s41598-017-07065-3

**Published:** 2017-07-28

**Authors:** Dorothée G. Drucker, Yuichi I. Naito, Stéphane Péan, Sandrine Prat, Laurent Crépin, Yoshito Chikaraishi, Naohiko Ohkouchi, Simon Puaud, Martina Lázničková-Galetová, Marylène Patou-Mathis, Aleksandr Yanevich, Hervé Bocherens

**Affiliations:** 1 Fachbereich Geowissenschaften, Forschungsbereich Paläobiologie, AG Biogeologie, Universität Tübingen, Hölderlinstr. 12, 72074 Tübingen, Germany; 2 Department of Biogeochemistry, Japan Agency for Marine-Earth Science and Technology, 2-15 Natsushima-cho, Yokosuka, 237-0061 Japan; 3 UMR 7194 (HNHP), MNHN/CNRS/UPVD, Sorbonne Universités, Institut de Paléontologie Humaine, 1 rue René Panhard, 75013 Paris, France; 4 UMR 7194 (HNHP), MNHN/CNRS/UPVD, Sorbonne Universités, Musée de l’Homme, Palais de Chaillot, 17 Place du Trocadéro, 75116 Paris, France; 5 Isotope Physiology, Ecology, and Geochemistry, Water and Material Cycles Division, Institute of Low Temperature Science, Hokkaido University, Kita-19, Nishi-8, Kita-ku, Sapporo, 060-0819 Japan; 6 Moravian Museum, Zelný trh 6, 65937 Brno, Czech Republic; 7 Hrdlička Museum of Man, Faculty of Science, Charles University, Viničná 7, 128 00 Praha, Czech Republic; 8 Department of Anthropology, Faculty of Philosophy and Arts, University of West Bohemia, Sedláčkova 15, 306 14 Pilsen, Czech Republic; 9 Institute of Archaeology, National Academy of Sciences of Ukraine, Heroiv Stalingrada 12, 04210 Kyiv, Ukraine; 10 Senckenberg Centre for Human Evolution and Palaeoenvironment (HEP), Universität Tübingen, Hölderlinstr. 12, 72074 Tübingen, Germany

## Abstract

Relatively high ^15^N abundances in bone collagen of early anatomically modern humans in Europe have often been interpreted as a specific consumption of freshwater resources, even if mammoth is an alternative high ^15^N prey. At Buran-Kaya III, access to associated fauna in a secured archaeological context and application of recently developed isotopic analyses of individuals amino acids offer the opportunity to further examine this hypothesis. The site of Buran-Kaya III is located in south Crimea and has provided a rich archaeological sequence including two Upper Palaeolithic layers, from which human fossils were retrieved and directly dated as from 37.8 to 33.1 ka cal BP. Results from bulk collagen of three human remains suggests the consumption of a high ^15^N prey besides the contribution of saiga, red deer, horse and hare, whose butchered remains were present at the site. In contrast to bulk collagen, phenylalanine and glutamic acid ^15^N abundances reflect not only animal but also plant protein contributions to omnivorous diet, and allow disentangling aquatic from terrestrial resource consumption. The inferred human trophic position values point to terrestrial-based diet, meaning a significant contribution of mammoth meat, in addition to a clear intake of plant protein.

## Introduction

Anatomically modern humans (AMHs) colonized Europe around 45-43 ka cal BP replacing Neanderthals after ca. 40 ka cal BP^1–3^ with potential cultural and/or biological interactions between these two human groups^4^. The exploitation by AMHs of a large diversity of ecosystems and food items is one of the highly debated scenarios to explain their successful expansion to the detriment of the possibly less flexible ecology of Neanderthal^5, 6^. The hypothesis of a broader dietary spectrum for AMH was fueled by observations of higher bone collagen ^15^N abundances in AMH compared with Neanderthals, which was interpreted as due to the addition of freshwater resources^7–9^ in contrast to a terrestrial-based diet typical of the Neanderthals^10–12^. However, the discrepancy in the location of the studied specimens, the overwhelming influence of meat in the protein contribution reflected in bulk collagen and non-systematic validation of the local stable isotope baseline may have contributed in oversimplifying the picture delivered by isotopic data^12^.

Several explanations can be evoked for high δ^15^N values in ancient human remains: a) high δ^15^N values in the terrestrial resources, especially in typical large herbivores whose meat provide the majority of dietary proteins over plants^13^, b) significant contribution of a given prey with higher δ^15^N values than the herbivores usually found in the archaeological sites, this resource being fish^7, 8^ or mammoth^13^. Freshwater fish is a ^15^N-enriched source of food compared with most of the terrestrial large herbivores, while their abundances in ^13^C are comparable^14^. On the other hand, Late Quaternary mammoth has been recognized as systematically ^15^N-enriched in comparison to other herbivores in the same environment, probably as the result of diet specialization^14^. The possible overlapping in ^13^C and ^15^N abundances between freshwater food and mammoth hinders accurate estimation solely from bulk collagen. Recent advances in stable nitrogen isotope analysis on individual amino acids should help disentangle the respective intake of resources from terrestrial and freshwater ecosystems^15, 16^ and may even reveal the contribution of plants to the human diet that is generally not detectable through bulk isotopic data^17, 18^. Phenylalanine (Phe) and glutamic acid (Glu) were specifically examined since the Phe ^15^N abundances reflect the local baseline (small trophic ^15^N-enrichment of 0.4 ± 0.4‰), while Glu ^15^N abundances depend not only on the baseline but also on the trophic position (large trophic ^15^N-enrichment of 8.0 ± 1.1‰)^19–21^. The difference in ^15^N of Glu-Phe (Δ^15^N_Glu-Phe_) is used to estimate the trophic position (TP) of a specimen (TP = 2 for herbivores, TP = 3 for primary carnivores, TP = 4 for secondary carnivores).

**Figure 1:**
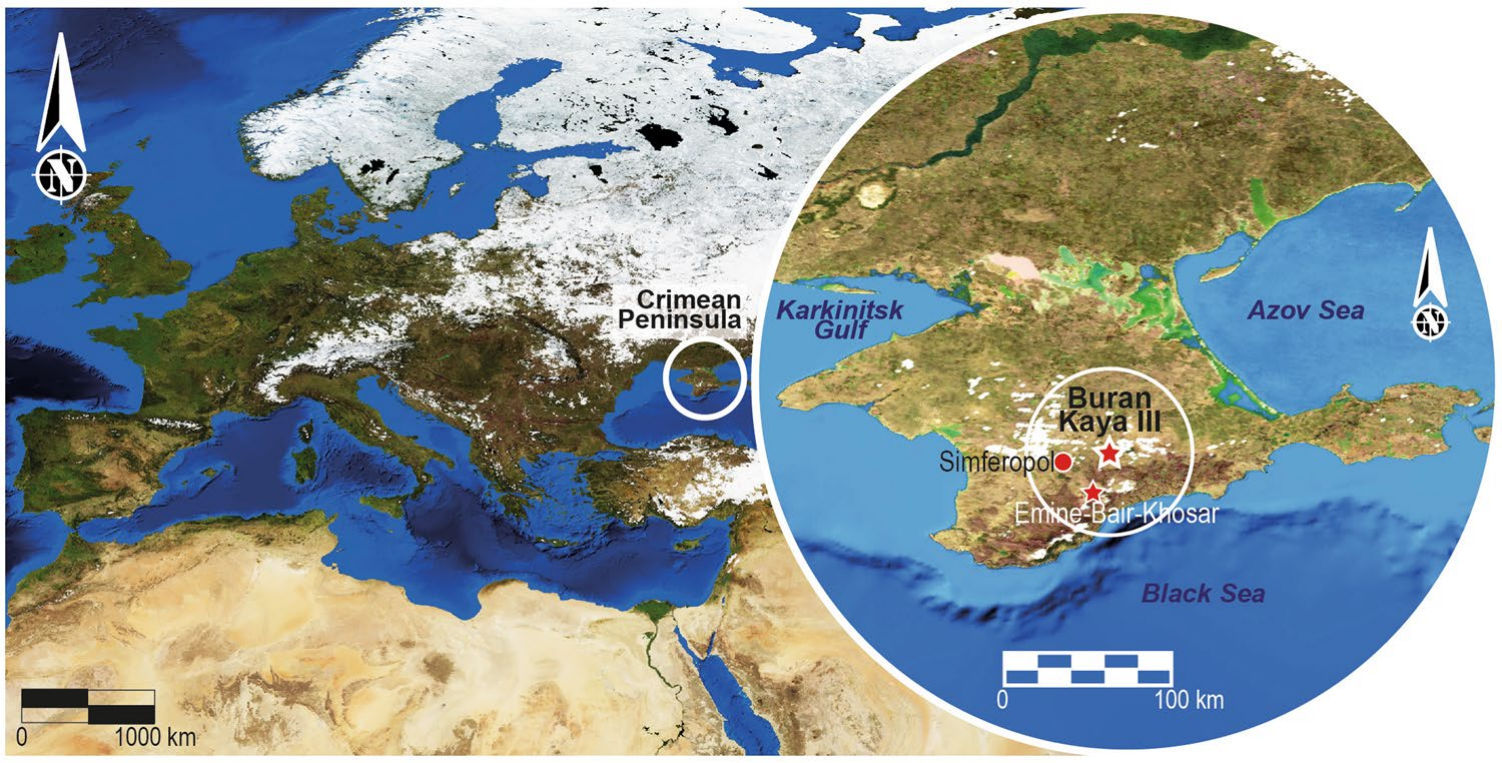
Current map of Europe with the location of Buran Kaya III site and Emine-Bair-Khosar cave in the Crimean Peninsula (map was designed by S. Puaud using open access NASA resources at http://eoimages.gsfc.nasa.gov/images/imagerecords/73000/73580/world.topo.bathy.200401.3×21600×21600.C1.jpg).

Here we consider the remains of AMHs found in Buran-Kaya III, a rock shelter located on the eastern bank of the Burulcha river in the Belogorsk region of south Crimea (Fig. 1; Supplementary Data 1, Supplementary Fig. 1). The site was discovered in 1990 by A. Yanevich (National Academy of Sciences of Ukraine) and excavated until 2001 by a team under the direction of A. Yanevich and A. Marks. The excavation was then resumed between 2009 and 2011 under the supervision of A. Yanevich and S. Péan. The stratigraphy of the site ranges from the Middle Palaeolithic to Neolithic with a complete sequence over the Middle to Upper Palaeolithic transition^22–26^ (Supplementary Fig. 2). Six Upper Palaeolithic archaeological layers, dated from 34.1 to 29.4 ka BP, i.e. 40.4 to 33.5 ka cal BP^26^ have provided a rich assemblage of lithic tools and faunal remains. In addition, bone tools, body ornaments (shells, mammoth ivory, red deer and fox teeth), and human fossils were retrieved from the Upper Palaeolithic layers 6-2 and 6-1 ranging from 32.5 to 29.4 ka BP (38.4-33.5 ka cal BP). Convergent palaeoecological results based on pollen, microfauna, large mammals and sediment studies points to a cold and dry climate during the establishment of layers of 6-2 and 6-1^26^. This corresponds to repeated and short occupations mainly devoted to the seasonal hunting of saiga antelopes during their summer migration^27^ (Supplementary Data 2). The human remains are currently the oldest AMHs from far south Eastern Europe^26, 28^. Anthropogenic modifications were observed in some of these human remains, which were not explained by dietary cannibalism^28, 29^ (Supplementary Data 3). This represents the oldest evidence of a complex treatment of the dead by anatomically modern humans in Eastern Europe.

This context offers an unique opportunity to investigate the diet of AMHs during the early phases of their expansion in Eastern and Central Europe. Human and animal specimens considered in this study come from layers 6-1 and 6-2, which were formerly attributed to the Gravettian culture^30, 31^. Direct AMS radiocarbon dating on the human remains demonstrated a human occupation ranging from 37.8 to 33.1 ka cal BP for these two layers^26, 28^. Preliminary data showed a higher difference than expected in bulk δ^15^N values between one of the dated human individual and two red deer samples of layer 6-1^28^.

We aim at verifying and identifying the prey causing the unusually high relative enrichment in ^15^N of the AMHs of Buran-Kaya III compared to their usual game species, namely saiga antelope, red deer and horse. We thus intend to test the relative probability that such a high ^15^N foodstuff would be of terrestrial – mammoth – rather than of aquatic – fish – origin through the ^15^N abundance on specific amino acids. The difference between the δ^15^N values of Glu and Phe will be especially examined to test how compatible is the trophic position (TP) deduced from the Δ^15^N_Glu-Phe_ with different theoretical combinations of terrestrial and aquatic dietary proteins.

## Results

We analysed directly dated human and animal specimens from layers 6-2 and 6-1 and an additional human from layer 6-1 for isotopic signature (Table 1). Moreover, we selected remains of saiga antelope (*Saiga tatarica*, n = 6), red deer (*Cervus elaphus*, n = 2), horse (*Equus* sp, n = 2), and hare (*Lepus* sp, n = 1) from layer 6-1 and 6-2. Small mammoth ivory fragments from layer 6-1 could be used since the reconstruction of a body ornament left a few pieces apart (*Mammuthus primigenius*, n = 1). Carnivores were represented by wolf (*Canis lupus*, n = 1) and fox (*Vulpes vulpes* or *Alopex lagopus*, n = 5). Fish remains are missing in the site despite systematic sieving of the sediment during the excavations. All the selected specimens provided well-preserved collagen, following established criteria^32, 33^, and were submitted to stable isotope analysis of bulk collagen (Table 2). All the collagen samples, except for one saiga and one horse specimen that could not be included due to technical constraints, were subsequently prepared for amino acid isotopic analysis.

**Table 1:** List of the sampled bone specimens from Buran-Kaya III and related radiocarbon dates.

Ref lab	Species	Sample	Layer	Excavation reference	^14^C BP	cal BP (95.4%)	^14^C source
BK3-07-01	*Homo sapiens*	cranial vault fgmt	6–1	2001 10Б (−155)	31,900 ± 240/220 GrA-37938	36,930-35,503	28
BK3-12-01	*Homo sapiens*	cranial vault fgmt	6–1	2001 10 A			
BK3-07-03	*Equus* sp.	lower cheek tooth R	6–1	2001 10Б			
BK3-07-04	*Cervus elaphus*	metacarpal fgmt	6–1	2001 9 A	31,320 ± 820 GifA-10021/SacA-19018	38,357-34,582	28
BK3-07-06	*Cervus elaphus*	tibia fgmt R	6–1	2001 9Б			
BK3-10-18	*Saiga tatarica*	tibia fgmt L	6–1	2009 9Z/272 (−146)			
BK3-08-04	*Saiga tatarica*	humerus fgmt L	6−1	2001 10 A			
BK3-07-02	*Saiga tatarica*	jawbone fgmt L	6−1	2001 10Б	31,530 ± 670 GifA-11216/SacA25133	37,735-34,749	26
BK3-07-05	*Saiga tatarica*	proximal phalanx	6−1	2001 9Б			
BK3-11-04	*Saiga tatarica*	radius fgmt L	6−1	2010 9Z/1054 (−146)	29,640 ± 170 GrA-53942/32,200 ± 450 OxA-25669	34,781-33,730 (combined)	26
BK3-08-01	*Lepus* sp.	humerus fgmt L	6−1	2001 10 A			
BK3-11-02	*Mammuthus primigenius*	processed ivory	6−1	2001 9 A (−152)			
BK3-07-08	*Canis lupus*	proximal phalanx	6−1	2001 11 A (−135)			
BK3-08-02	cf. *Vulpes vulpes*	tibia fgmt R	6−1	2001 10 A			
BK3-08-03	*Vulpes* sp.*/Alopex lagopus*	femur fgmt L	6−1	2001 10 A			
BK3-07-07	*Vulpes* sp.*/Alopex lagopus*	metatarsal V L	6−1	2001 11Б			
BK3-11-01	*Homo sapiens*	cranial vault fgmt	6–2	2001 10Б	32450 ± 250/230 GrA-50457	37,831–36,450	26
BK3-08-05	*Equus* sp.	metatarsal fgmt R	6–2*	2001 9Б	34050 ± 260/2s40 GrA-40485/34910 ± 950 GifA-80181/SacA-12260	40,078–38,508 (combined)	28
BK3-10-19	*Saiga tatarica*	metacarpal fgmt R	6–2	2009 9Z/636 (−159)	29440 ± 190/180 GrA-50460	34,643–33,486	26
BK3-08-07	cf. *Alopex lagopus*	humerus fgmt R	6–2	2001 10Б			
BK3-08-09	cf. *Alopex lagopus*	ulna fgmt L	6–2	2001 9 A			

Excavation reference corresponds to year square/object, field number and depth in cm; fgmt stands for fragment, L for left and R for right. *Indicates that the former stratigraphy position is now questioned based on the direct radiocarbon date (possible re-attribution to 6–3^26^).

**Table 2:** Results of stable isotope analyses of collagen (δ^13^C_coll_, δ^15^N_coll_, δ^15^N_Phe_, δ^15^N_Glu_) from the animal and human samples of Buran-Kaya III.

Ref lab	Species	Sample	Layer	C_coll_	N_coll_	C:N_coll_	δ^13^C_coll_	δ^15^N_coll_	δ^15^N_Phe_	δ^15^N_Glu_	TP	TP
				(%)	(%)		(‰)	(‰)			C3	Aqua
BK3-07-01	*Homo sapiens*	cranial vault fgmt	6−1	43.2	15.3	3.3	−19.4	15.4	17.4	21.2	2.6	1.1
BK3-12-01	*Homo sapiens*	cranial vault fgmt	6−1	41.9	15.4	3.2	−18.9	16.8	17.6	20.9	2.5	1.0
BK3-07-03	*Equus* sp.	lower cheek tooth R	6−1	20.6	7.3	3.3	−20.4	8.1	13.1	11.8	1.9	
BK3-07-04	*Cervus elaphus*	metacarpal fgmt	6−1	43.0	15.0	3.3	−19.1	7.9	13.3	10.6	1.8	
BK3-07-06	*Cervus elaphus*	tibia fgmt R	6−1	24.8	8.6	3.4	−19.1	10.3	12.2	11.6	2.0	
BK3-10-18	*Saiga tatarica*	tibia fgmt L	6−1	39.5	14.1	3.3	−17.0	11.2	13.3	12.4	2.0	
BK3-08-04	*Saiga tatarica*	humerus fgmt L	6−1	42.0	14.8	3.3	−16.4	11.8	16.3	15.5	2.0	
BK3-07-02	*Saiga tatarica*	jawbone fgmt L	6−1	34.9	12.0	3.4	−15.6	9.5	14.5	12.9	1.9	
BK3-07-05	*Saiga tatarica*	proximal phalanx	6−1	45.7	15.6	3.4	−16.4	10.2	13.9	13.1	2.0	
BK3-11-04	*Saiga tatarica*	radius fgmt L	6−1	45.7	16.0	3.3	−15.5	10.0				
BK3-08-01	*Lepus* sp.	humerus fgmt L	6−1	41.0	14.3	3.4	−20.7	6.0	12.5	9.9	1.8	
BK3-11-02	*Mammuthus primigenius*	processed ivory	6−1	39.6	14.1	3.3	−20.7	12.6	17.5	15.5	1.8	
BK3-07-08	*Canis lupus*	proximal phalanx	6−1	37.1	13.1	3.3	−18.7	13.0	17.4	24.7	3.1	
BK3-08-02	cf. *Vulpes vulpes*	tibia fgmt R	6−1	38.6	14.0	3.2	−17.2	11.5	12.3	15.8	2.6	
BK3-08-03	*Vulpes* sp.*/Alopex lagopus*	femur fgmt L	6−1	42.2	15.0	3.3	−17.2	13.5	13.5	17.7	2.6	
BK3-07-07	*Vulpes* sp.*/Alopex lagopus*	metatarsal V L	6−1	44.4	15.2	3.4	−17.9	9.4	14.2	18.9	2.7	
BK3-11-01	*Homo sapiens*	cranial vault fgmt	6−2	34.6	13.2	3.1	−18.8	15.8	16.6	19.7	2.5	1.0
BK3-08-05	*Equus* sp.	metatarsal fgmt R	6−2*	32.2	11.5	3.3	−19.2	9.0				
BK3-10-19	*Saiga tatarica*	metacarpal fgmt R	6−2	41.8	14.7	3.3	−17.8	10.7	16.1	14.0	1.8	
BK3-08-07	cf. *Alopex lagopus*	humerus fgmt R	6−2	42.6	14.8	3.4	−19.2	14.2	15.7	20.3	2.7	
BK3-08-09	cf. *Alopex lagopus*	ulna fgmt L	6−2	36.2	12.8	3.3	−19.6	7.8	11.7	15.7	2.6	

The carbon and nitrogen composition of the collagen is given through elemental composition (C_coll_, N_coll_) and atomic ratio (C:N_coll_). fgmt stands for fragment, L for left and R for right, *indicates that the former stratigraphy position is now questioned based on the direct radiocarbon date (possible re-attribution to 6–3^26^).

### Environment and diet reconstruction based on bulk collagen

The animal samples can be divided into two groups based on a cluster analysis of their δ^13^C_coll_ values (Supplementary Fig. 3). The first group exhibited δ^13^C_coll_ values ranging from −20.7 to −18.7‰ and included red deer, horse, hare and mammoth for the herbivorous species, as well as the wolf of layer 6-1 and the two fox specimens of layer 6-2. The second group corresponded to δ^13^C_coll_ values ranging from −17.9 to −15.5‰ and encompassed all the saiga and fox samples of layer 6-1, suggesting a predation relationship between the two species.

The δ^15^N_coll_ values of the fauna exhibited a high variability. Red deer and horse δ^15^N_coll_ values varied from 7.9 to 10.3‰, while hare showed a lower value of 6.0‰ and saiga provided among the highest δ^15^N_coll_ values (9.5 to 11.8‰). Finally, the mammoth ivory displayed the highest δ^15^N_coll_ values with 12.6‰, reaching a comparable range as the wolf (13.0‰). The range of δ^15^N_coll_ values of the fox specimens from layer 6-1 with relatively low δ^13^C_coll_ values was even wider (7.8 to 14.2‰) than for the specimens in the second group with higher δ^13^C_coll_ values from layer 6-2 (9.4 to 17.2‰). Each group corresponded not only to different layers but also possibly to different species (polar fox or red fox). If the foxes of layer 6-1 could clearly have consumed saiga, the foxes of layer 6-2 reflected a drastically different diet. Low abundances in both ^13^C and ^15^N can be explained by the specialized hunting of hare, while low ^13^C with high ^15^N abundances points to a large proportion of larger herbivore (red deer and/or horse) meat.

**Figure 2:**
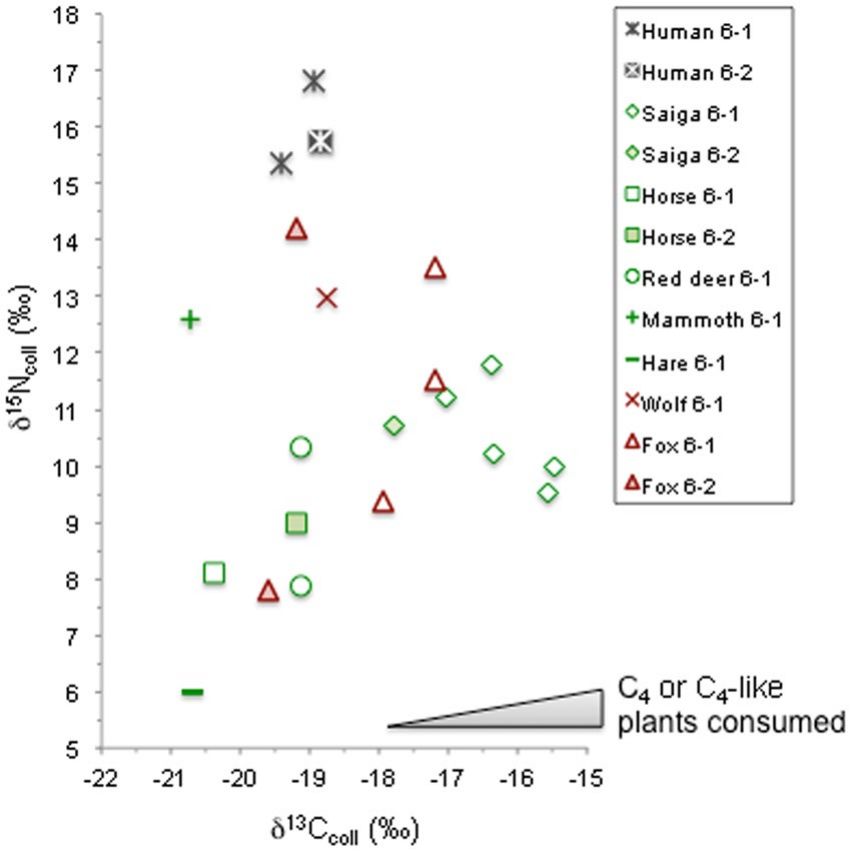
Measured δ^13^C_coll_ and δ^15^N_coll_ values of saiga, horse, red deer, mammoth, hare as herbivores (in green) and wolf, fox and anatomically modern humans from layers 6-1 and 6-2 of Buran-Kaya III (in red).

The human individuals had δ^13^C_coll_ and δ^15^N_coll_ values ranging from −19.4 to −18.8‰ and from 15.4 to 16.8‰, respectively (Fig. 2). Their δ^15^N_coll_ values were thus at least 1‰ higher than the highest values measured in fox or wolf. This suggests the consumption of prey with higher ^15^N abundances than those accessible to the animal predators. The δ^13^C_coll_ on the other hand implies the intake of animals mainly dependent on C_3_ plants, and thus does not fit with a dominant saiga-based protein diet. Beyond the single value available for Buran-Kaya III, the mammoth is a species known to display such low δ^13^C_coll_ for relatively high δ^15^N_coll_ values^10–14^, and should be considered as a potential meat contributor to human diet. On another hand, the freshwater resource contribution cannot be ruled out, even if no fish remains have been retrieved from the site, despite water sieving of the excavated sediments.

### Diet and trophic position reconstruction based on collagen amino acids

The analysis of compound-specific δ^15^N values of phenylalanine and glutamic acid serves as a way of detection of possible aquatic contribution in human diet^15, 34^. The δ^15^N_Phe_ values of herbivores and carnivores reflect mainly those of the primary producers, at the base of the food chain, due to limited trophic ^15^N-enrichment^19–21^. The δ^15^N_Phe_ values of the herbivores of Buran-Kaya III reflected the observed pattern of the δ^15^N_coll_ with even less overlap between mammoth with the highest value of 17.5‰ and the other herbivores, such as red deer, horse and hare with the lowest values of 12.2 to 13.3‰, and saiga with intermediate values (13.3 to 16.3‰) (Fig. 2). This confirms that the high δ^15^N_coll_ value of the mammoth is linked to the specificity of its diet rather than to physiological traits^18, 35, 36^. The δ^15^N_Phe_ values of the foxes (11.7 to 15.7‰) were relatively consistent with those of the saiga, as expected from their comparable δ^13^C_coll_ values. An exception was the fox specimen of 6-2 with low δ^15^N_coll_ value and one individual of 6-1 since both exhibited δ^15^N_Phe_ values low enough to be comparable to the group of red deer, horse and hare. These foxes had possibly less access to saiga meat than their counterparts. The wolf and the human individuals of layer 6-1 exhibited δ^15^N_Phe_ values from 17.4 to 17.6‰ that were similar to mammoth (17.4‰), suggesting this large game animal being a significant source of dietary protein. The human sample of layer 6-2 provided a slightly lower value of 16.6‰, which could reflect a lower influence of mammoth meat in the diet and a slightly higher contribution of saiga as source of animal protein.

The calculated trophic positions (TPs) of the herbivores using TP(C_3_) equation were ranging from 1.8 to 2.0 and averaged at 1.9, slightly lower than the theoretical value for herbivores, which was found at the early Upper Palaeolithic site of Scladina in Belgium (mean TP = 2, excluding cave bears)^18^. The TP position of the wolf at 3.1 instead of 3 also reflects a possible uncertainty of ± 0.1 in the calculation of the TP value and/or variability in its diet. Interestingly, the foxes’ TP values ranged from 2.6 to 2.7, which is consistent with the omnivorous and opportunistic habits reported for this small canid^37, 38^.

**Figure 3:**
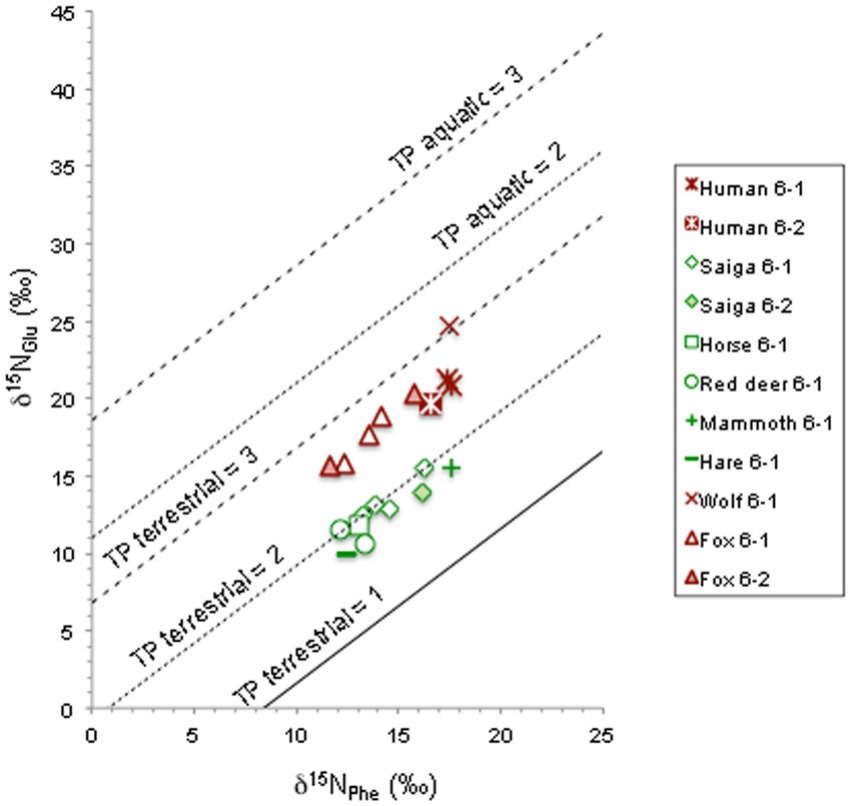
Measured δ^15^N_Phe_ and δ^15^N_Glu_ values on saiga, horse, red deer, mammoth, hare as herbivores and wolf, fox and human remains from layers 6-1 and 6-2 of Buran-Kaya III. Solid, dotted, and dashed lines indicate theoretical lines for δ^15^N_Phe_ and δ^15^N_Glu_ values of organisms with TP (Trophic Position) in terrestrial ecosystem = 1, 2, and 3, and aquatic ecosystem = 2 and 3, respectively.

The calculation of TPs of the human individuals analysed at Buran-Kaya III gave values of 2.5 and 2.6 based on TP(C_3_) equation, while 1.0 and 1.1 values were obtained using TP(Aqua) equation (Table 2; Fig. 3). If the first result can be interpreted as a terrestrial-based diet with a clear intake of plant protein, the second results would place the human as the same trophic level as aquatic plants, which is unrealistic. In order to test different scenarios including aquatic resources in addition to terrestrial foodstuffs, we have tested a linear model comparing the δ^15^N_Glu_ measured for the analysed humans with the δ^15^N_Glu_ values calculated from the measured δ^15^N_Phe_ at different end-points^15^ (Supplementary Data 4, Supplementary Fig. 4) for consumption of : aquatic primary consumers (TP(Aqua) = 3), aquatic secondary consumers (TP(Aqua) = 4), terrestrial plants (TP(C_3_) = 2), terrestrial primary consumers (i.e. herbivore; TP(C_3_) =3), and a 50:50 mix of terrestrial plant and herbivores (TP(C_3_) = 2.5). The estimations, with an error of ±10%, should be considered as qualitatively indicative, but underline that the input of plants is necessary (Table 3). The aquatic contribution could go above 10% only in the case that no herbivore meat would be consumed, which is not consistent with the local archaeological evidence of animal hunting by hunter-gatherer at that time. In case of half of the dietary protein being provided by plants, which involves more than half of the diet due to the lower nitrogen content of plant tissues against animal meat, the possible contribution of aquatic resources remains theoretically very low. The intake of human meat due to cannibalism practices has been questioned at Buran-Kaya III due to the occurrence of cut marks testifying to scalping and disarticulation processes, even if the comparison with the human modification on animal remains favours the hypothesis of mortuary practice or ritual cannibalism rather than dietary canibalism^28, 29^ (Supplementary Data 3). A regular consumption of human meat would have increased the TP over the value of carnivores (TP = 3). The significant consumption of human meat can thus be ruled out for the analysed individuals of Buran-Kaya III.

**Table 3:** Quantitative evaluation of freshwater resource consumption for the human individuals of Buran-Kaya III.

BK3-07-01	TP(Aqua) = 3	TP(Aqua) = 4
TP(C3) = 2	24.0	17.3
TP(C3) = 2.5	5.5	3.7
TP(C3) = 3	n/a	n/a
**BK3-12-01**	**TP(Aqua) = 3**	**TP(Aqua) = 4**
TP(C3) = 2	20.8	15.0
TP(C3) = 2.5	1.6	1.1
TP(C3) = 3	n/a	n/a
**BK3-11-01**	**TP(Aqua) = 3**	**TP(Aqua) = 4**
TP(C3) = 2	20.1	14.4
TP(C3) = 2.5	0.6	0.4
TP(C3) = 3	n/a	n/a

Indicated percentages correspond to the contribution of either primary consumers (TP(Aqua) = 3) or secondary consumers (TP(Aqua) = 4) from aquatic ecosystem to the human diet in association to terrestrial plants (TP(C_3_) = 2), or terrestrial primary consumers (TP(C_3_) = 3), or a 50:50 mix of terrestrial plant and herbivores (TP(C_3_) = 2.5). These calculations are indicative since the values of the end-members are based on the theoretical TP lines. n/a (non applicable) stands for unsolvable results.

## Discussion

In general, the δ^13^C_coll_ and δ^15^N_coll_ values of the large herbivores from Buran-Kaya III are higher than those observed in other early Upper Palaeolithic mammoth steppe ecosystems^11, 39^. This difference, in addition to the C_4_-like plant consumption by the saiga antelope, testifies to the higher aridity of the Crimean context compared with northwest Europe^40, 41^. Compared with other herbivores of the site, the saiga diet included plants with a higher δ^13^C values than those expected in C_3_ plants^42^. Buran-Kaya III is currently located in a premontane forest-steppe area without favourable conditions for C_4_ plants development^43^. From an isotopic point of view, saigas of Buran-Kaya III are comparable with some of the most ^13^C-enriched modern specimens of Kazahkstan, which are known to consume significant amounts of Chenopodiaceae^44^ (Supplementary Data 5, Supplementary Fig. 5). The C_4_-like or C_3_-C_4_ intermediate photosynthesis mechanisms of the Chenopodiaceae linked to adaptations to arid environments can explain the high δ^13^C values of the ancient Crimean saiga. Interestingly, the equation based on C_3_ terrestrial vascular plants provided sensitive TP values for all the saiga specimens independently of their δ^13^C_coll_ values (Supplementary Data 6 and Table 1). The likely access to desert and salty environments for this migrating species contrasts with the local C_3_ environment reflected by the horse and red deer of the site. At the extreme points of the variability in herbivore δ^15^N_coll_ values, the relative low ^15^N abundance of hares fits with previous findings of a ^15^N-depleted signature for lagomorphs in contrast to coeval larger herbivores^45, 46^. On the other hand, the high δ^15^N_coll_ value of the mammoth sample derived from an ornament artefact is consistent with the ^15^N-enriched signature (ca. 4‰ difference with horse/red deer) typical of this species^14^, while no other mammoth remains have been found in layers 6-1 and 6-2, which could be explained by the type of activities conducted at the site and carcasses transport decisions^11^. Indeed, the occupation of Buran-Kaya III was highly seasonal and the human activity was devoted to butchery of small and middle-size mammals during repeated short episodes. Interestingly, only saiga carcasses were brought complete among the middle-size herbivores, while the skeletons of the other species, such as red deer, are partially represented which could reflect far distance hunting. Mammoth meat procurement by humans found at the site would have happened out of the context of the occupation of the site of Buran-Kaya III. Interestingly, only 24 km southwest, a natural accumulation at Emine-Bair-Khosar cave in the Crimean mountains delivered remains of mammoth in a context dated around 38.7-36.6 ka cal BP (level H, 33,500 ± 400 BP)^47^. Thus, living mammoth could have been encountered in Crimea until the early phases of the Upper Palaeolithic. However, the exact dating of mammoth survival in the neighbouring Buran-Kaya III is hindered by the lack of sites of references in the area, probably accentuated by the loss of territories north and west of the current peninsula due to the rise of the sea-level since the Last Glacial Maximum^24, 48^.

The fox samples could be separated into two categories based on the δ^13^C_coll_ values: one with values higher than −18‰ (layer 6-1), and a second with values lower than −19‰ (layer 6-2). The δ^15^N_Phe_ values of the foxes of layer 6-1 generally fit with those of the saiga, except for one individual whose low value makes the saiga contribution difficult to discriminate from the red deer/horse and hare meat intake. The low δ^13^C_coll_ and δ^15^N_coll_ of one fox of layer 6-2 could be interpreted as a high consumption of hare, which is consistent with its low δ^15^N_Phe_ value. The other fox specimen of layer 6-2 shows a potential important access to large herbivores carcasses. The consumption of saiga antelope could be mainly the result of scavenging, since the fox only occasionally preys on this species, especially on calves, in modern ecosystems^44, 49^ and traces of small carnivores gnawing was found on some saiga bone remains at Buran-Kaya III. The potential contributions of red deer/horse, saiga or hare as prey of the wolf are wide and largely overlapping based on the SIAR model using bulk collagen isotopic composition, hindering a more precise reconstruction of the diet composition (Supplementary Fig. 6). The mammoth however appears as a significant source of food of up to 30% of the diet of the wolf (Supplementary Data 7), which is consistent with the similar δ^15^N_Phe_ values between wolf and mammoth ivory.

**Figure 4:**
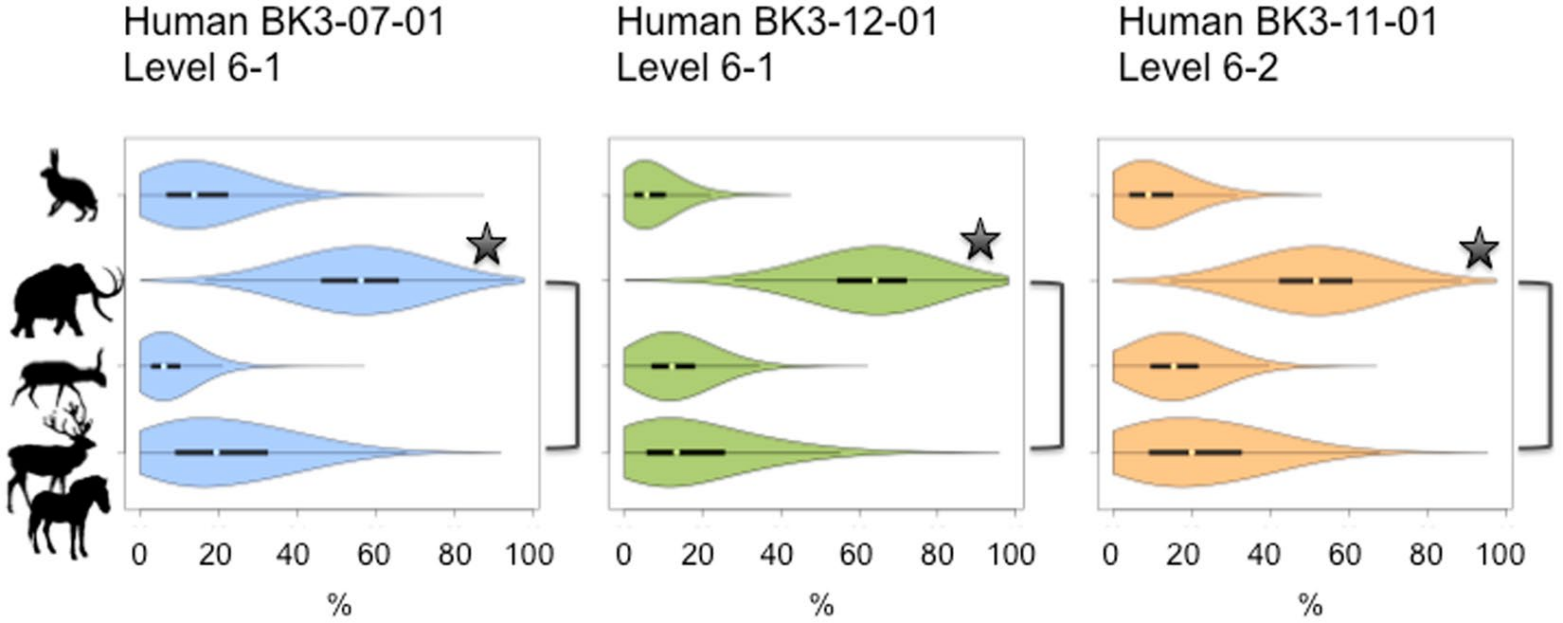
Proportional contribution of Deer&Horse (red deer and horse), Saiga (saiga antelope), Mammoth (woolly mammoth) and Hare (hare) (from bottom to top along the y axis) as estimated by SIAR using the considering both δ^13^C_coll_ and δ^15^N_coll_ values for human remains from Buran-Kaya III layers 6-2 and 6-1. Black boxes and whiskers show the median with 1^st^ and 3^rd^ quartiles and ranges with 1.5 times length of the interquartile range above the 3^rd^ quartile or below the 1^st^ quartile, respectively. The shaded area indicates the Kernel density plot of the probability density of prey proportions. The brackets link the resources with a significant negative correlation in their posterior distribution. Stars are placed close to the food resource whose significant contribution is in accordance with the δ^15^N_Phe_ values.

**Figure 5:**
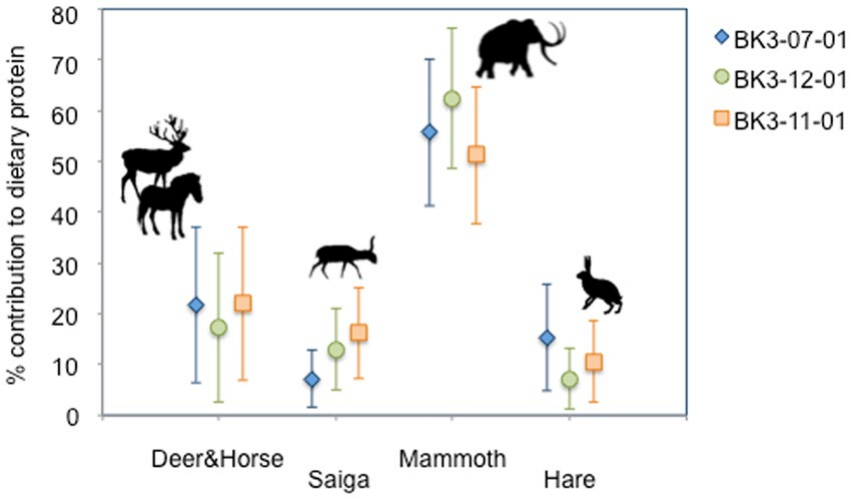
Proportional contribution of Deer&Horse (red deer and horse), Saiga (saiga antelope), Mammoth (woolly mammoth) and Hare (hare) as estimated by SIAR model for human remains from Buran-Kaya III layers 6-2 and 6-1. Each symbol corresponds to the mean protein diet contribution to a given human individual. Bars indicate standard deviations.

The TP values of the humans of Buran-Kaya III argue in favour of terrestrial-based subsistence practices, with no significant consumption of freshwater foodstuffs (Supplementary Fig. 7). Hence, the mammoth represents the most likely protein source contributing to the high δ^15^N values in human collagen and could account for up to 40–50% of the meat protein. In contrast, saiga, the main prey hunted on a seasonal basis at Buran-Kaya III, shows a potential protein contribution to human diet lower than 20%. Interestingly, a lower δ^15^N_Phe_ value in association with a slightly higher δ^13^C_coll_ value compared with those of the human samples of 6-1 points to a higher intake of saiga antelope for the individual of 6-2. Possible red deer and horse contributions appear comparable if not slightly higher than for saiga. Finally, hare appears as a protein source used to a limited extent by the analysed humans with a maximum of 15% of protein contribution with the highest probability density according to the SIAR Bayesian model (Figs 4 and 5). Such isotopic reconstructions are not only qualitative but also highly difficult to compare directly with the relative amounts of species remains left at the site since collagen reflect several years if not decades of meat intake at the scale of an individual, while the faunal accumulation results from seasonal activities of a group. Moreover, such comparison would require a conversion of the number of remains in relative meat weight, taking into account the large contrast between megaherbivores such as mammoth, and medium to small herbivores, such as saiga and hare. A contribution of up to ca. 20% of plant to the dietary proteins was estimated for late Neanderthals from Spy (Belgium)^18^ with TPs values between 2.7 and 2.8. Our estimation for the modern humans of Buran-Kaya III suggests a possible higher consumption of plants, which is consistent with the higher availability of such resources in more southern latitudes.

The individuals of Buran-Kaya III provide the highest δ^15^N_coll_ values reported so far for east and central Europe during the early Upper Palaeolithic^9^. A diet incorporating freshwater fish was proposed for other individuals with high ^15^N from comparable chronological context, such as Oase 1 (13.3‰)^50^ as well as Muierii 1 and 2 (12.3 and 12.4‰ respectively)^51^, and Cioclovina 1 from Romania (12.7‰)^51^. Despite limited sample size, the case of Buran-Kaya III, with its exceptional archaeological context and good collagen preservation, shows that the mammoth could be the source of such high ^15^N signal and suggests that it should be more systematically considered as an alternative explanation to aquatic resources. Isotopic studies of western European late Neanderthals also point to the significant consumption of mammoth as well^11, 39^. Thus, the role of mammoth in human subsistence during the early Upper Palaeolithic should be further examined in future research.

## Methods

### Sample preparation for isotopic analyses

Specimens were chosen from taxonomically and anatomically identified compact bone pieces. Collagen was extracted following previously established protocol^52, 53^. The extraction process includes a step of soaking in 0.125 M NaOH between the demineralization and solubilization steps to achieve the elimination of lipids and humic acids.

### Isotope analyses of bulk collagen

Elemental analysis (C_coll_, N_coll_) and isotopic analysis (δ^13^C_coll_, δ^15^N_coll_) were conducted at the Department of Geosciences of Tübingen University using a NC2500 CHN-elemental analyzer coupled to a Thermo Quest Delta+ XL mass spectrometer. The standard, internationally defined, is a marine carbonate (PDB) for δ^13^C and atmospheric nitrogen (AIR) for δ^15^N. Analytical error, based on within-run replicate measurement of laboratory standards (albumen, modern collagen, USGS 24, IAEA 305 A), was ±0.1‰ for δ^13^C values and ±0.2‰ for δ^15^N values. Reliability of the δ^13^C_coll_ and δ^15^N_coll_ values can be established by measuring its chemical composition, with C/N_coll_ atomic ratio ranging from 2.9 to 3.6^32^, percentage of C_coll_ and N_coll_ above 8% and 3%^33^, respectively.

Cluster analyses using Ward’s minimum variance method were performed on stable carbon and nitrogen isotopic composition, with the software SAS JMP version 12.2.0.

### Isotope analyses of individual amino acids

All amino acid samples were prepared following the established protocols^51^. The bone and ivory collagen samples were subjected to hydrolysis by 12 N HCl at 110 °C for 12 h, followed by derivatisation with thionyl chloride/2-propanol (1:4, v/v) at 110 °C for 2 h and pivaloyl chloride/dichloromethane (1:4, v/v) at 110 °C for 2 h. The nitrogen isotopic compositions of the individual amino acid derivatives were measured by gas chromatography/combustion/IRMS (GC/C/IRMS) using an Agilent Technology 6890 GC (Agilent) coupled to a Thermo Finnigan Delta^plus^XP IRMS (Thermo Fisher Scientific, Waltham, MA, USA) via combustion and reduction furnaces. Instrumental analysis was performed according to previous methods, with a few modifications of the equipment settings^54^. Standard mixtures of nine amino acids with known δ^15^N values were injected into the GC/C/IRMS every five runs to confirm the reproducibility of the isotope measurements. The mean accuracy and precision of the reference mixtures were 0.0‰ and 0.4–0.7‰ (mean of 1σ), respectively.

The characteristic Δ^15^N_Glu-Phe_ value is −8.4‰ for most wild terrestrial plants and 3.4‰ for aquatic - marine and freshwater – plants. Based on these empirical estimations^21, 55^, equations were established to calculate the trophic position (TP) in terrestrial C_3_ and aquatic food webs. For C3-plant-based ecosystems: TP(C3) = [(Δ^15^N_Glu-Phe_ + 8.4)/7.6] + 1. For aquatic ecosystems: TP(Aqua) = [(Δ^15^N_Glu-Phe_ − 3.4)/7.6] + 1.

### SIAR Bayesian model

The relative contribution of the different prey to the average diet of the human individuals was simulated using a Bayesian mixing model approach performed in the Stable Isotope Analysis in R (SIAR) package^56^, using the R software version 3.3.0^57^. SIAR offers the possibility to work with multiple sources and to incorporate uncertainty in input data, yielding not only a range of possible dietary proportions, but providing also their relative probability distribution^52^. The relative contributions of four groups of preys as sources of animal protein were tested using the SIAR model: red deer/horse, saiga, mammoth and hare. Mean and standard deviations were calculated for use in the model (Supplementary Table 2). In the case of a single individual, as for mammoth and hare, we attributed a standard deviation value that was calculated from other datasets measured on archaeological context (Supplementary Tables 3 and 4). We considered a trophic enrichment factor (TEF) of +1.1 ± 0.2‰ and +3.8 ± 1.1‰ for δ^13^C and δ^15^N values of bulk collagen, respectively^58^. The food categories to be tested through SIAR model were defined as follow: deer and horse altogether (Deer&Horse), saiga antelope (Saiga), woolly mammoth (Mammoth) and hare (Hare).

## Electronic supplementary material

Supplementary Information
