# Supplementary material for: Isotopic analyses suggest mammoth and plant in the diet of the oldest anatomically modern humans from far southeast Europe
Source: Sci Rep. 2017 Jul 28;7:6833. doi: 10.1038/s41598-017-07065-3 (PMC5533724; doi:10.1038/s41598-017-07065-3)
Supplement: Supplementary file 1 — Supplementary Information [file 41598_2017_7065_MOESM1_ESM.pdf]

Isotopic analyses suggest mammoth and plant in the diet of the oldest anatomically modern humans from far southeast Europe

SUPPLEMENTARY INFORMATION

Dorothée G. Drucker[1,10*], Yuichi I. Naito[2*], Stéphane Péan[3], Sandrine Prat[4], Laurent Crépin[3], Yoshito Chikaraishi[5], Naohiko Ohkouchi[2], Simon Puaud[4], Martina Lázničková-Galetova[3,6,7,8], Marylène Patou-Mathis[3], Aleksandr Yanevich[9], Hervé Bocherens[1,10]

1 Fachbereich Geowissenschaften, Forschungsbereich Paläobiologie, AG Biogeologie, Universität Tübingen, Hölderlinstr. 12, 72074 Tübingen, Germany

2 Department of Biogeochemistry, Japan Agency for Marine-Earth Science and Technology, 2-15 Natsushima-cho, Yokosuka 237-0061, Japan

3 UMR 7194 (HNHP), MNHN/CNRS/UPVD, Sorbonne Universités, Institut de Paléontologie Humaine, 1 rue René Panhard, 75013 Paris, France

4 UMR 7194 (HNHP), MNHN/CNRS/UPVD, Sorbonne Universités, Musée de l'Homme, Palais de Chaillot, 17 Place du Trocadéro, 75116 Paris, France

5 Isotope Physiology, Ecology, and Geochemistry, Water and Material Cycles Division, Institute of Low Temperature Science, Hokkaido University, Kita-19, Nishi-8, Kita-ku, Sapporo 060-0819, Japan

6 Moravian Museum, Zelný trh 6, 65937 Brno, Czech republic

7 Hrdlička Museum of Man, Faculty of Science, Charles University, Viničná 7, 128 00 Praha, Czech republic

8 Department of Anthropology, Faculty of Philosophy and Arts, University of West Bohemia, Sedláčkova 15, 306 14 Pilsen, Czech Republic

9 Institute of Archaeology, National Academy of Sciences of Ukraine, Heroiv Stalingrada 12, 04210 Kyiv, Ukraine
10 Senckenberg Centre for Human Evolution and Palaeoenvironment (HEP), Universität Tübingen, Hölderlinstr. 12, 72074 Tübingen, Germany
*These authors contributed equally to this work. Correspondence and requests for materials should be addressed to D.G.D (email: dorothee.drucker@ifu.uni-tuebingen.de) or Y.I.N. (email: ynaito@jamstec.go.jp)

Supplementary Data 1

Geographical and geological settings of Buran-Kaya III

The Buran-Kaya III site is located in the South of Crimea, 30 km at the ENE of Simferopol on the North slope of the Crimean Mountains (Supplementary Fig. 1). Discovered in 1990 by A. Yanevich and excavated until 2001[1], it was investigated again through new fieldworks between 2009 and 2011, focusing on 9 archaeological layers attributed to the Middle and Upper Palaeolithic (Supplementary Fig. 2b), from bottom to top: Streletskaya or eastern Szeletian (layer C), Micoquian (layer B), Aurignacian (layers 6–5, 6–4, 6–3), Gravettian *sensu lato* (layers 6–2, 6–1, 5–2), and Final Palaeolithic (layer 4). The Middle Palaeolithic layer (Micoquian) is strikingly situated between an Early Upper Palaolithic layer (Streletskaya or eastern Szeletian) and three layers attributed to the Aurignacian.

This rock shelter is situated at the foot of an escarpment of Cretaceous limestone. The heterogeneity of this geological formation permits, under periglacial conditions, differential erosion of the limestone strata, and the formation of a gelifraction balm.

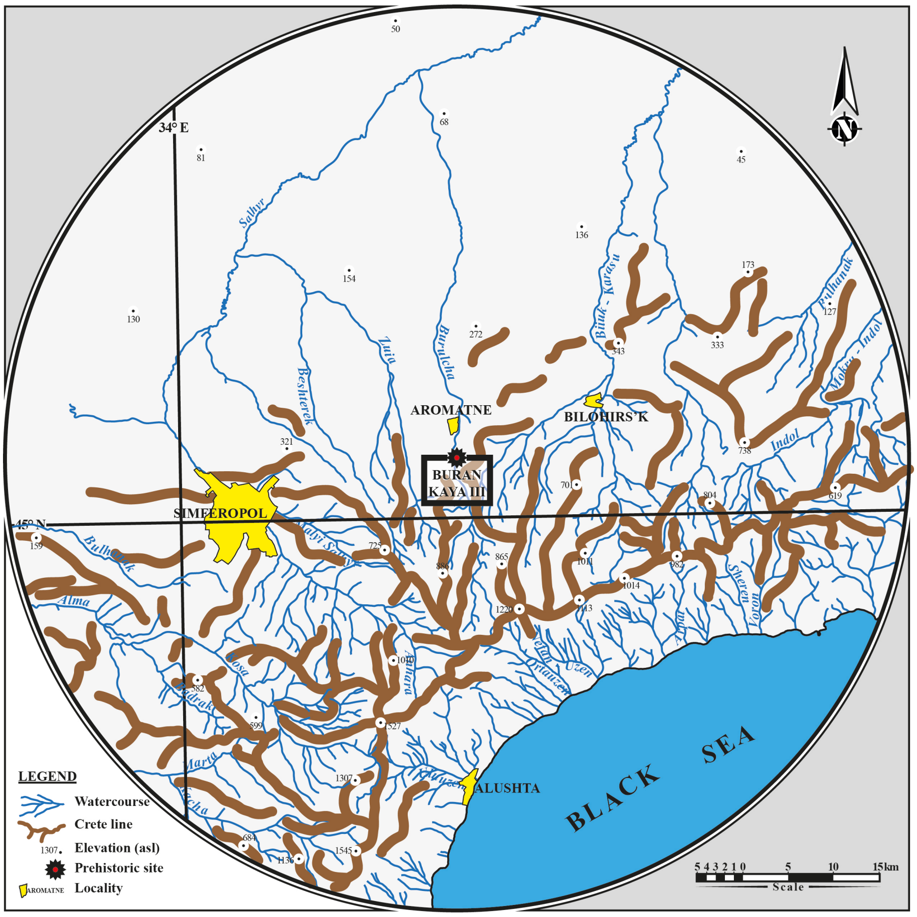

Supplementary Figure 1. Water system 50km around the prehistoric site of Buran Kaya III. (map was designed by S. Puaud using open access NASA resources at http://eoimages.gsfc.nasa.gov/images/imagerecords/73000/73580/world.topo.bathy.200401.3x21600x21600.C1.jpg).

Stratigraphy and sedimentological interpretation

The rock shelter of Buran-Kaya III (Supplementary Fig. 2a) contains a stratified infilling composed of ten layers (two sedimentary units divides by a silt clay level) (Supplementary Fig. 2b). The main sedimentary facies is diamicton. The coarse fraction is provided by frost shattering of the limestone walls. The matrix is mainly silty or silty sand with grain size and sorting typically of an eolian sediment. Beneath the silt clay level, sediments of the lower unit (layers C to 6-2) are coarser and less well-sorted than the sediments of the upper unit (layers 6-1 to 5-2) (Supplementary Fig. 2c). The climato-sedimentary interpretation of this sequence indicates, for the lower unit, contrasting climatic conditions with cold and wet winters, followed by less severe but still moist periods with

runoff into the shelter. In the upper unit (from silt clay level to 5-2), seasonal contrasts are less pronounced and eolian deposits are dominant; the climatic conditions become significantly drier than below. Layer 4 is more organic and coarser than below, which are characteristics of contrasting conditions (frost shattering, pedobiological activities).

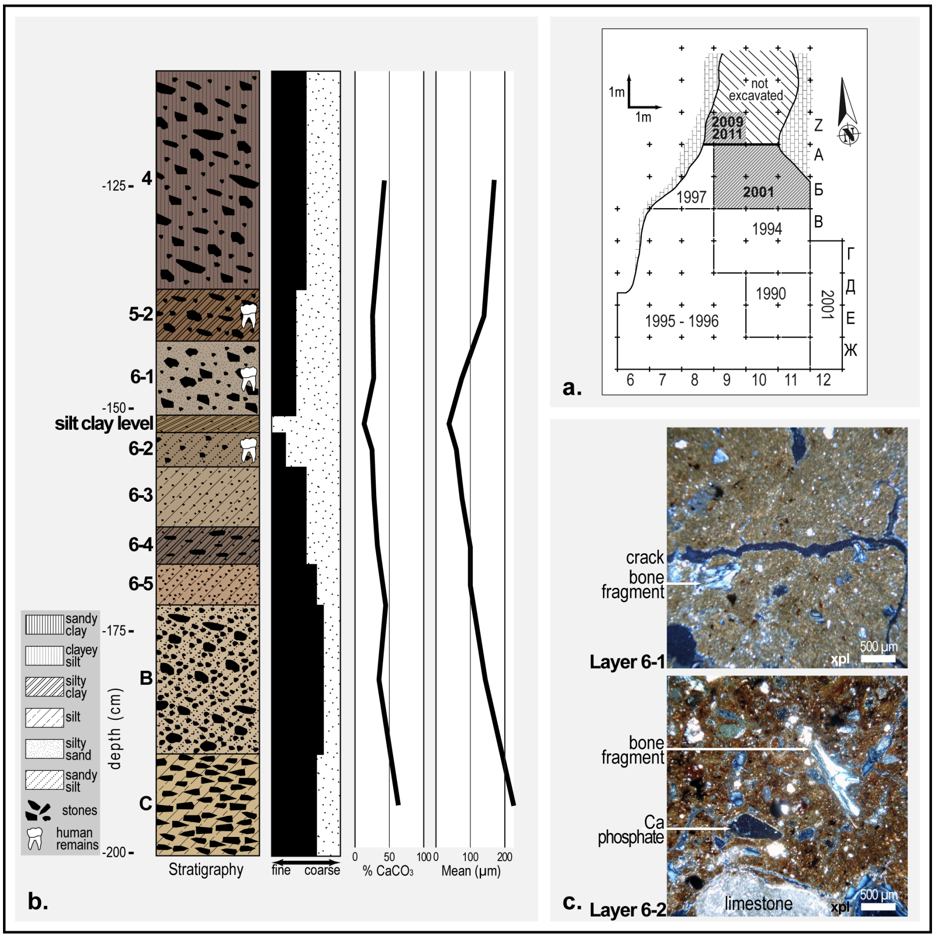

Supplementary Figure 2. Buran-Kaya III (Crimea). a. Plan of the rock shelter with the location of the different excavation areas. b. Synthetic stratigraphic column with results of sedimentological analysis (grain-size and Ca carbonate rate). c. Microscopic pictures with details of the texture and elements of the layers 6-2 and 6-1).

Supplementary Data 2

Faunal remains

According to zooarchaeological analyses[2], the mammal remain assemblages from the Gravettian *sensu lato* layers (6-2, 6-1 and 5-2) show a predominance of saiga antelope *(Saiga tatarica)*, red and polar foxes (*Vulpes vulpes* and *Alopex lagopus*), and hare (*Lepus* sp.).

During both 2001 and 2009-2011 fieldworks, the most abundant layer, 6-1, yielded a total of 117,351 faunal remains, of which less than 2% were anatomically and taxonomically identified: total Number of Identified Specimens NISP=2,213; total Minimum Number of individuals by combined parameters[3] (Poplin, 1983) cMNI=40. The faunal assemblage shows a predominance of saiga antelopes (43% of NISP, cMNI=15), mostly represented by adult individuals, red and polar foxes (29.5% of NISP, cMNI=7) and hares (13% of NISP, cMNI=5). The other determined mammals are equids (*Equus* sp., 10% of NISP, cMNI=3*)*, bovines (*Bison* sp. or *Bos primigenius*, 4% of NISP, cMNI=2), carnivores (*Canis lupus, Ursus* sp., *Felis silvestris* and Mustelids, 1% of NISP, cMNI=5), cervids (*Cervus elaphus* and *Rangifer tarandus,* 0.4% of NISP, cMNI=2) and woolly rhinoceros (*Coelodonta antiquitatis*, one specimen, 0.1% of NISP, cMNI=1).

Mammoth remains are only represented by a few processed artefacts made of ivory (bead and engraved plate). Other osseous artefacts are composed of 40 bone pointed tool fragments, with debris from *in situ* production, 1 perforated fox canine and 35 perforated mollusc shells.

Taphonomical studies show a limited modification of bone surfaces by carnivores, whose tooth marks are only observed on 1% of the NISP. Almost all skeletal elements of saiga antelope, fox and hare are represented but axial and girdle parts are relatively scarce. The bone material is very fragmented.

A high proportion of burned bones were found (25% of the total number of remains), resulting from intense combustion activities produced by humans. Cut marks and fracture features due to human activities were observed on 7% of the identified specimens, belonging to six taxa: saiga antelope, hare, bovine, horse, fox and wolf. Human modifications of the saiga bone surfaces (on cranial and postcranial remains) mainly reflect disarticulation and defleshing activities, breakage and marrow extraction. Consumption of hares is also evidenced by taphonomic modifications. Foxes also

exhibit marks of disarticulation and defleshing, and also traces of fur removal on metapodials and tarsals.

Indices of seasonality tend to indicate a summer mortality of the saiga antelopes, which represent the main game of the hunter-gatherers in Buran-Kaya III. The subsistence activities in the studied area of the site, i.e. the rear part of the rock-shelter, seem relatively modest and do not exhibit the complete butchery *chaîne opératoire*. Only skinning, filleting and marrow extraction activities were identified. The first stages of butchery probably took place elsewhere, in another location or even another site. Furthermore, there is a high usage of bone as fuel within the site, which could be explained by a very cold environmental context with rare plant resources and/or by activities of preparation of meat and fat broth.

These zooarchaeological results from the Gravettian *s.l.* layers suggest short-term and recurrent settlements. The site of Buran-Kaya III would have been used as a typical temporary hunting camp, with more intense and/or long activities in layer 6-1 than 6-2.

Supplementary Data 3

The anatomically modern humans of Buran-Kaya III

More than two hundred human remains were discovered in three well-documented Upper Palaeolithic layers (6-2, 6-1, 5-2), attributed to the Gravettian *sensu lato* technocomplex. Layer 6-1 has yielded the richest assemblage in terms of number of remains, minimum number of individuals and anatomical parts. These human remains are highly fragmented and consist mostly of cranial parts and teeth (90% of the remains), whereas the postcranial skeleton is barely represented (hand phalanx fragments). Based on the age determination from the dental remains, at least 5 individuals belonging to three developmental age groups (juvenile, subadult and adult) can be identified in this layer. The health of these individuals was good[4], with no caries. Enamel dental hypoplasia was observed only in the youngest individuals at the beginning of their childhood (~3 years). Based on the combination of morphological features on the occipital and on the dental remains, the human

remains from Buran-Kaya III are distinguished from Neanderthal and are attributed to Anatomically modern humans[5].

Among the fragmented human remains (n=172) from layer 6-1, 8 % exhibits human modifications such as cut marks. Their morphology and their position in respect to muscular insertions suggest that scalping and disarticulation processes occurred. Three possible anthropogenic actions on human bodies can be hypothesized to explain the occurrence of these cut marks on several human remains: mortuary practice, dietary and non-dietary cannibalism. Comparative taphonomical analyses (e.g. spatial repartition of the remains, skeletal representation and surface bone modifications) between the human remains and the saiga antelope, which is the main game according to the archaeozoological analyses, show that these two taxa were not processed in a similar way. Furthermore human skulls were intentional selected. Consequently, the hypothesis of a dietary cannibalism[6] is not supported by these data. Specific mortuary practices (such as post-mortem disarticulation processes of corpses for secondary disposal) or ritual cannibalism are proposed as alternative scenarios[5,7].

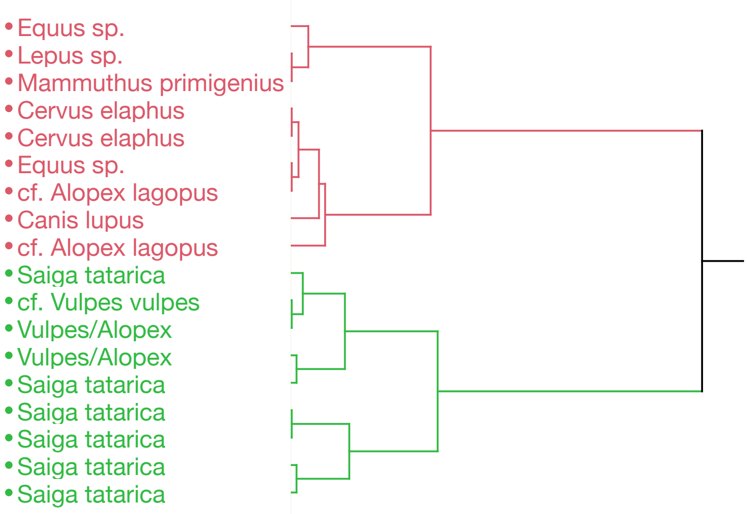

Supplementary Figure 3. Cluster analysis of the animal specimens of Buran-Kaya III based on their $\delta^{13}C_{coll}$ values.

Supplementary Data 4

Quantification of aquatic resource consumptions by humans using distances between TP(C3) and TP (Aqua) lines and $\delta^{15}N_{Glu}$ and $\delta^{15}N_{Phe}$ value of humans

An example of the model calculating the percentage of freshwater resources in the diet of the human individuals of Buran-Kaya III is given in Supplementary Figure 3 below. For the human from layer 6-2, different values of $\delta^{15}N_{Glu}$ can be inferred from the measured $\delta^{15}N_{Phe}$ value for diet based only on terrestrial primary producers (TP(C3)=2), or on terrestrial primary consumers (TP(C3)=3), or on aquatic primary consumers (TP(Aqua)=3), or on aquatic secondary consumers (TP(Aqua)=4). Then these end-members $\delta^{15}N_{Glu}$ values can be compared with the measured $\delta^{15}N_{Glu}$ of the individual from layer 6-2 following a linear model. Note that the calculated percentages of different end-members contributing to the diet would not be affected by a change in the slope of the mixing lines since the four TP lines are always parallel.

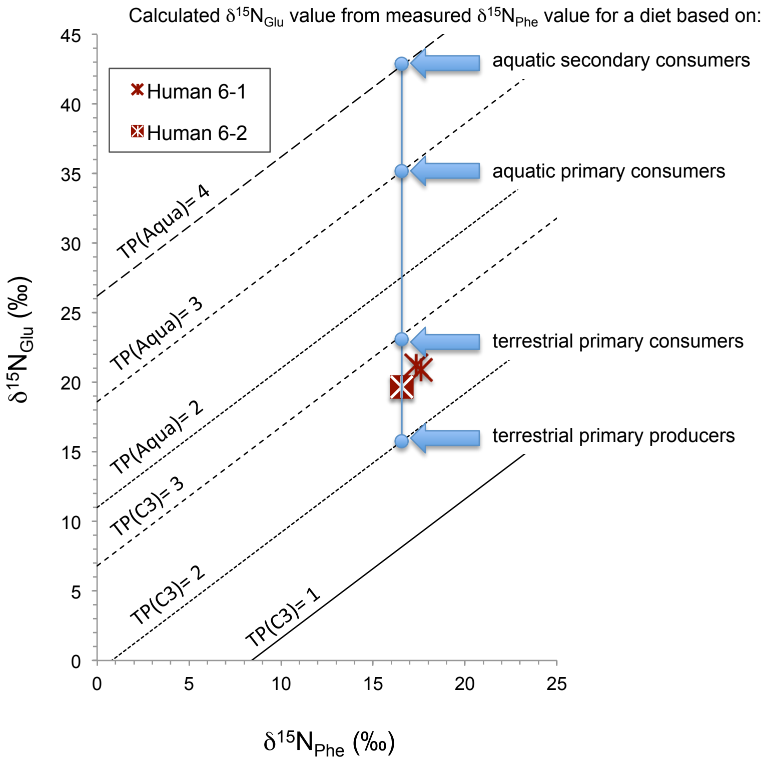

Supplementary Figure 4. Example of the mixing line (in blue) for the individual of layer 6-2 and indication of the $\delta^{15}N_{Glu}$ corresponding to different end-members (blue arrows).

Example of calculation of $\delta^{15}N_{Glu}$ values for different terrestrial and aquatic end-members for human individual BK3-11-01:

Based on the equation $TP(C3) = [(\delta^{15}N_{Glu} - \delta^{15}N_{Phe} + 8.4)/7.6] + 1$,

Calculated $\delta^{15}N_{Glu} = (7.6^{*} TP(C_3)) +$ measured $\delta^{15}N_{Phe} - 16$, which leads to a value of 15.8 for TP(C3)= 2 and of 23.4 for $TP(C_3)= 3$

Based on the equation $TP(Aqua) = [(\delta^{15}N_{Glu} - \delta^{15}N_{Phe} - 3.4)/7.6] + 1$,

Calculated $\delta^{15}N_{Glu} = (7.6^{*} TP(C_3)) +$ measured $\delta^{15}N_{Phe} - 4.2$, which leads to a value of 35.2 for TP(Aqua)= 3 and of 42.8 for TP(Aqua)= 4

Then, the calculation to estimate the relative contribution of the different end-members is as the following example considering a diet based on terrestrial plant and meat:

Based on the equation Measured $\delta^{15}N_{Glu}$ = (Plant Proportion x Calculated $\delta^{15}N_{Glu}$ for $TP(C_3)$ =2) + (Meat Proportion x Calculated $\delta^{15}N_{Glu}$ for TP(C3) =3), with Plant Proportion = 1- Meat Proportion,

Plant Proportion = (Measured $\delta^{15}N_{Glu}$ – 23.4) / (Calculated $\delta^{15}N_{Glu}$ for $TP(C_3)$ =2 - Calculated $\delta^{15}N_{Glu}$ for TP(C3) =3)

Supplementary Data 5

Isotopic comparison of the saiga antelope of Buran-Kaya III and modern saiga populations

When compared to modern saiga population[8], the saiga antelopes of Buran-Kaya III exhibit the same relatively high $\delta^{15}N_{coll}$ values expected for a species living in dry conditions (Supplementary Fig. 4). Their $\delta^{13}C_{coll}$ values are more comparable to those of the modern specimens from western Mongolia, meaning higher values than those of the modern groups from central Kazhakstan and southwestern Russia (Volga region). Since the saiga antelopes of Kazhakstan consume C_4- like plants[9], we tentatively interpret the increase in $\delta^{13}C_{coll}$ values as reflecting an increased aridity.

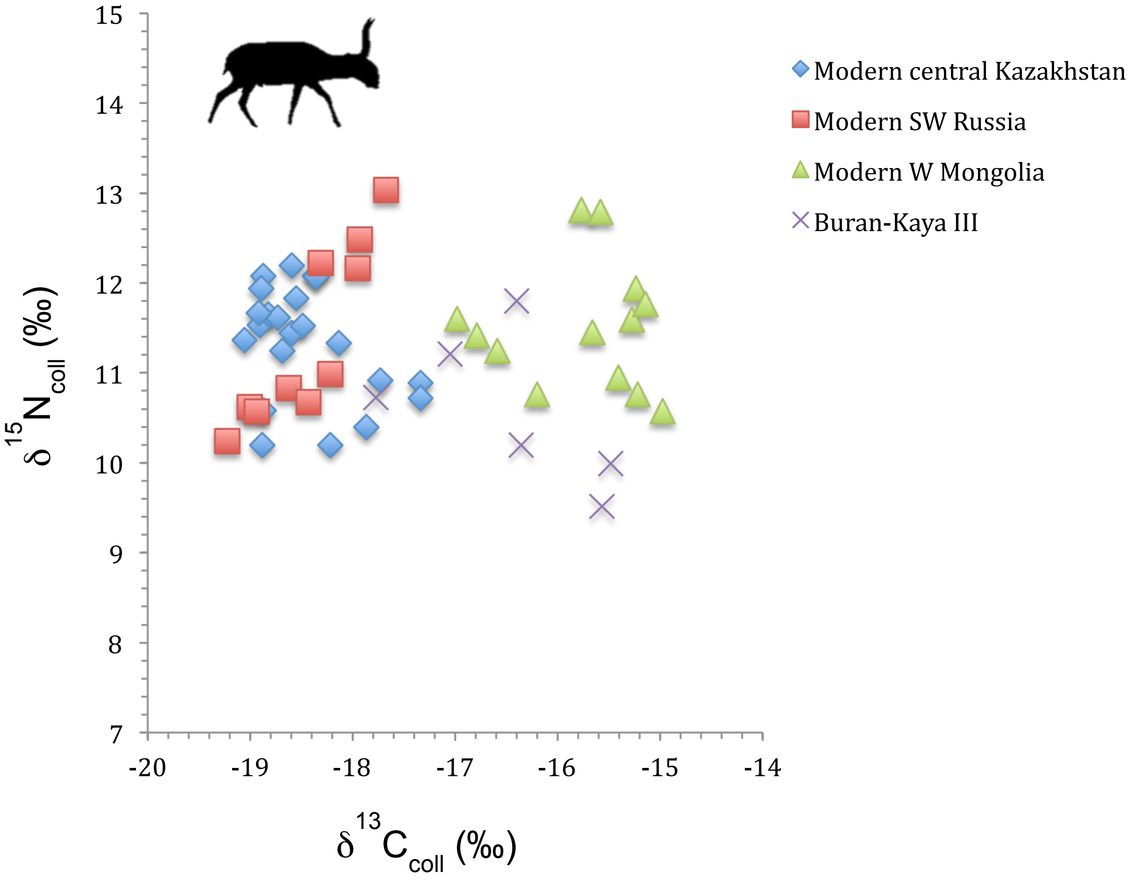

Supplementary Figure 5. Comparison of the $\delta^{13}C_{coll}$ and $\delta^{15}N_{coll}$ values of the saiga antelopes from Buran-Kaya III and those of modern specimens from central Kazhakstan, southwestern (SW) Russia and western (W) Mongolia. Data for modern specimens, obtained on hair samples, were converted into collagen values[10] and corrected for the change in $\delta^{13}C$ values of atmospheric CO_2 during the post-industrial time period (reference value: -6.4‰[11] using an established equation[12]) (animal drawing by D. Drucker).

Supplementary Data 6

Saiga antelope of Buran-Kaya III and trophic position calculation based on TP(C_3) and TP(C_4)

As for the other herbivores of Buran-Kaya III, the trophic position (TP) of the saiga antelopes was calculated based on the equation established in terrestrial C_3 ecosystems[13,14]:

$TP(C3) = [(\Delta^{15}N_{Glu\text{-}Phe} + 8.4)/7.6] +1$

Another equation was determined for C_4 ecosystems[13,14] as follows:

$$TP(C4) = [(\Delta^{15}N_{Glu\text{-}Phe} - 0.4)/7.6] + 1$$

The relatively high $\delta^{13}C_{coll}$ values of the saiga at Buran-Kaya III could imply the consumption of C_3-C_4 intermediate or C_4- like plants. However, when we applied the TP(C_4) equation to the saiga, the results ranged from 0.7 to 0.8 (Supplementary Table 1), meaning TP values not high enough for reaching the plant trophic level. Obviously, the TP(C_3) should be applied to all herbivores, including the saiga antelope. The reasons the TP(C_4) equation is inadequate for the saiga antelopes of Buran-Kaya III could either be due to (1) the lack of "true" C_4 plant in their diet, or (2) to the fact that this equation is based on ecosystems with cultivated C_4 plants.

Supplementary Table 1. Results of stable isotope analyses of individual amino acids ($\delta^{15}N_{Phe}$ and $\delta^{15}N_{Glu}$) of saiga antelopes from Buran-Kaya III. The trophic position (TP) was calculated using the equation established for C_3 ecosystems and C_4 ecosystems.

Lab N°	Species	Sample	Level	Reference	$\delta^{15}N_{Phe}$	$\delta^{15}N_{Glu}$	TP C3	TP C4
BK3-10-18	*Saiga tatarica*	tibia L	6-1	2009 6-1c	13.3	12.4	2.0	0.8
BK3-08-04	*Saiga tatarica*	humerus L	6-1	2001 6-1.2	16.3	15.5	2.0	0.8
BK3-07-02	*Saiga tatarica*	jawbon L	6-1	2001 6-1.5	14.5	12.9	1.9	0.7
BK3-07-05	*Saiga tatarica*	phalanx L	6-1	2001 6-1.5	13.9	13.1	2.0	0.8

Supplementary Data 7

Results of the SIAR reconstruction for the animal carnivores based on bulk isotopic ratios

The food categories to be tested through the SIAR model were defined as follow: deer and horse altogether (Deer&Horse), saiga antelope (Saiga), woolly mammoth (Mammoth) and hare (Hare). For each category, we considered the average and standard-deviation of the $\delta^{13}C_{coll}$ and $\delta^{15}N_{coll}$ values of the specimens without distinction between level 6-1 and 6-2 (Supplementary Table 2). In the case of a single individual, as for mammoth and hare, we attributed a standard-deviation value that was calculated from other datasets measured on archaeological context (Supplementary Tables

3 and 4). In both cases, we selected the highest values obtained, which were to ± 0.4‰ and ±0.9‰ for mammoth (Maisières-Canal dataset) and ±0.5‰ and ±0.8‰ for hare (Combe-Saunière 1 dataset), corresponding to the standard-deviation of the $\delta^{13}C_{coll}$ and $\delta^{15}N_{coll}$ value respectively.

Supplementary Table 2. Results of stable isotope analyses of collagen ($\delta^{13}C_{coll}$, $\delta^{15}N_{coll}$) of the herbivores of level 6-1 and 6-2 of Buran-Kaya III: The carbon and nitrogen composition of the collagen is given through elemental composition (C_{coll}, N_{coll}) and atomic ratio ($C:N_{coll}$).

Lab N°	Species	Sample	Level	Reference	C_{coll} (%)	N_{coll} (%)	$C:N_{coll}$	$\delta^{13}C_{coll}$ (‰)	$\delta^{15}N_{coll}$ (‰)
BK3-07-03	*Equus* sp.	premolar/molar R inf	6-1	2001 6-1.5	20.6	7.3	3.3	-20.4	8.1
BK3-08-05	*Equus* sp.	metatarsal R	6-2	2001 6-2.1	32.2	11.5	3.3	-19.2	9.0
BK3-07-04	*Cervus elaphus*	metacarpal	6-1	2001 6-1.5	43.0	15.0	3.3	-19.1	7.9
BK3-07-06	*Cervus elaphus*	tibia R	6-1	2001 6-1.5	24.8	8.6	3.4	-19.1	10.3
							**Av**	**-19.5**	**8.8**
							**SD**	**0.6**	**1.1**
BK3-10-18	*Saiga tatarica*	tibia L	6-1	2009 6-1c	39.5	14.1	3.3	-17.0	11.2
BK3-08-04	*Saiga tatarica*	humerus L	6-1	2001 6-1.2	42.0	14.8	3.3	-16.4	11.8
BK3-07-02	*Saiga tatarica*	jawbon L	6-1	2001 6-1.5	34.9	12.0	3.4	-15.6	9.5
BK3-07-05	*Saiga tatarica*	phalanx L	6-1	2001 6-1.5	45.7	15.6	3.4	-16.4	10.2
BK3-11-04	*Saiga tatarica*	radius L	6-1	2010 6-1	45.7	16.0	3.3	-15.5	10.0
BK3-10-19	*Saiga tatarica*	metacarpal R	6-2	2009 6-2c	41.8	14.7	3.3	-17.8	10.7
							**Av**	**-16.4**	**10.6**
							**SD**	**0.9**	**0.8**

Supplementary Table 3. Results of stable isotope analyses of collagen ($\delta^{13}C_{coll}$, $\delta^{15}N_{coll}$) of bone and ivory of mammoth (*Mammuthus primigenius*) from the Middle Paleolithic site of Lynford Quarry (Norfolk, United Kingdom) and the Gravettian site of Maisières-Canal (Belgium). The carbon and nitrogen composition of the collagen is given through elemental composition (C_{coll}, N_{coll}) and atomic ratio ($C:N_{coll}$).

Site	Lab N°	Material	Reference	C_{coll} (%)	N_{coll} (%)	$C:N_{coll}$	$\delta^{13}C_{coll}$ (‰)	$\delta^{15}N_{coll}$ (‰)	Source
Lynford Quarry, Norfolk	LQ3	bone	51143	41.4	13.6	3.5	-22.1	7.4	15
Lynford Quarry, Norfolk	LQ4	bone	50368	45.2	15.0	3.5	-22.3	6.9	15
Lynford Quarry, Norfolk	LQ5	bone	50289	43.6	15.3	3.3	-21.9	6.0	15
Lynford Quarry, Norfolk	LQ11	bone	51530	44.2	15.2	3.4	-21.8	7.6	15

Lynford Quarry, Norfolk	LQ14	bone	51348	40.9	14.1	3.4	-22.0	6.0	15
Lynford Quarry, Norfolk	LQ22	bone	51928	44.5	15	3.4	-21.9	6.9	15
Lynford Quarry, Norfolk	LQ23	bone	51971	39.0	13.4	3.4	-22.4	7.2	15
Lynford Quarry, Norfolk	LQ26	skull bone	unstratified	36.3	11.9	3.6	-22.2	6.7	15
Lynford Quarry, Norfolk	LQ12	tusk ivory	51615	40.2	13.6	3.4	-22.4	7.8	15
Lynford Quarry, Norfolk	LQ17	tusk ivory	51706	36.4	11.8	3.6	-22.2	7.2	15
Lynford Quarry, Norfolk	LQ25	tusk ivory	51952	44.2	15.4	3.3	-21.1	8.6	15
Lynford Quarry, Norfolk	LQ27	tusk ivory	51303	42.9	14.7	3.4	-22.0	7.8	15
Lynford Quarry, Norfolk	LQ28	tusk ivory	51303	44	14.6	3.5	-22.3	9.3	15
Lynford Quarry, Norfolk	LQ29	tusk ivory	51979	44.7	15.6	3.3	-22.1	7.6	15
Lynford Quarry, Norfolk	LQ33	tusk ivory	51775	40.8	13.2	3.6	-21.7	8.6	15
						**Av**	**-22.0**	**7.4**	
						**SD**	**0.3**	**0.9**	
Maisières-Canal, Belgium	OxA-18008	cut rib	nd	42.1	15.3	3.2	-21.4	8.3	16
Maisières-Canal, Belgium	OxA-18009	gnawed carpal	J 11	42.9	15.2	3.3	-21.7	7.6	16
Maisières-Canal, Belgium	OxA-17946	tusk ivory	J/K 10	40.5	14.8	3.2	-20.7	9.7	16
Maisières-Canal, Belgium	OxA-17947	tusk ivory	J/K 10	40.5	14.8	3.2	-20.8	9.6	16
Maisières-Canal, Belgium	OxA-17962	ivory artefact	K 10	43.1	15.2	3.3	-21.0	9.4	16
						**Av**	**-21.1**	**8.9**	
						**SD**	**0.4**	**0.9**	

Supplementary Table 4. Results of stable isotope analyses of collagen ($\delta^{13}C_{coll}$, $\delta^{15}N_{coll}$) of hare (*Lepus timidus*) from the Solutrean level of Combe Saunière 1 (Dordogne, France) and the Magdalenian level of Kesslerloch (Schaffhausen, Switzerland). The carbon and nitrogen composition of the collagen is given through elemental composition (C_{coll}, N_{coll},) and atomic ratio ($C{:}N_{coll}$).

**Site**	**Lab N°**	**Material**	**Reference**	**Level**	**C_{coll} (%)**	**N_{coll} (%)**	**$C{:}N_{coll}$**	**$\delta^{13}C_{coll}$ (‰)**	**$\delta^{15}N_{coll}$ (‰)**	**Source**
CombeSaunière 1, France	CS4100	femur L	H18A 33 (3)	IVb	41.7	15.4	3.2	-20.5	1.8	17
CombeSaunière 1, France	CS4200	femur L	H16B 38(2)	IV	42.7	15.4	3.2	-20.7	3.2	17
CombeSaunière 1, France	CS4300	femur L	H21C 72(5)	IVb	42.7	15.8	3.2	-20.0	2.7	17
CombeSaunière 1, France	CS4400	femur L	I18 9043	IV	42.3	15.6	3.2	-19.6	1.4	17
							**Av**	**-20.2**	**2.3**	
							**SD**	**0.5**	**0.8**	
Kesslerloch, Switzerland	KSL-19	tibia L	HEII:147	KSLH II	37.5	14.1	3.1	-20.8	0.9	18
Kesslerloch, Switzerland	KSL-20	tibia L	HLGK:167	KSLH GK	39.6	14.5	3.2	-20.2	0.7	18
Kesslerloch, Switzerland	KSL-21	tibia L	HLGK:167	KSLH GK	38.8	14.1	3.2	-20.4	-0.3	18
Kesslerloch, Switzerland	KSL-22	tibia L	HLGK:167	KSLH GK	40.3	14.2	3.3	-20.8	0.5	18
Kesslerloch, Switzerland	KSL-23	tibia L	HE21:330	KSLH IIc	38.4	15.0	3.0	-21.4	0.2	18
Kesslerloch, Switzerland	KSL-24	tibia L	HE21:330	KSLH IIc	38.1	14.9	3.0	-20.6	0.3	18

Kesslerloch, Switzerland	KSL-25	tibia L	HE21:330	KSLH IIc	40.7	14.6	3.3	-20.4	2.3	18
Kesslerloch, Switzerland	KSL-26	tibia L	HEZD:169	KSLH II/III	35.5	13.7	3.0	-19.7	1.3	18
Kesslerloch, Switzerland	KSL-27	tibia L	HEZD:169	KSLH II/III	37.2	12.8	3.4	-20.2	0.4	18
Kesslerloch, Switzerland	KSL-28	tibia L	HEZD:169	KSLH II/III	35.8	12.8	3.3	-20.4	0.6	18
Kesslerloch, Switzerland	KSL-29	tibia L	HEZD:169	KSLH II/III	37.3	14.2	3.1	-20.5	1.1	18
Kesslerloch, Switzerland	KSL-30	tibia L	HEZD:169	KSLH II/III	35.2	13.4	3.1	-20.2	0.5	18
Kesslerloch, Switzerland	KSL-31	tibia L	HE33:600	KSLH IIIAs	37.3	14.6	3.0	-20.1	1.9	18
Kesslerloch, Switzerland	KSL-32	tibia L	HE33:600	KSLH IIIAs	38.0	14.5	3.1	-20.4	1.0	18
Kesslerloch, Switzerland	KSL-33	tibia L	HE33:600	KSLH IIIAs	38.0	14.7	3.0	-20.3	1.6	18
Kesslerloch, Switzerland	KSL-34	tibia L	HE33:600	KSLH IIIAs	30.4	12.0	2.9	-20.2	0.9	18
Kesslerloch, Switzerland	KSL-35	tibia L	HE33:600	KSLH IIIAs	40.6	14.3	3.3	-20.9	1.5	18
							**Av**	**-20.4**	**0.9**	
							**SD**	**0.4**	**0.7**	

For each predator specimen (carnivore and human), the possible contribution of each food source was evaluated using SIAR and considering the combined $\delta^{13}C_{coll}$ and $\delta^{15}N_{coll}$ values. Then, the relative contributions of the food categories as an animal protein source were tested and the results expressed in terms of relative probability. Some food categories exhibited significant negative correlations in their posterior distribution. It means that a higher proportion of one food category involved a lower proportion of the other. This was the case between Deer&Horse and Hare as protein source for all the animal predators. In some cases, the $\delta^{15}N_{Phe}$ values could help to favour one of the correlated food category over the other (stars in Supplementary Fig. 5). Following this reasoning, a large contribution of saiga for the fox specimen 08-03 of level 6-1 and of mammoth for the fox specimen 08-07 of level 6-2) was indeed demonstrated.

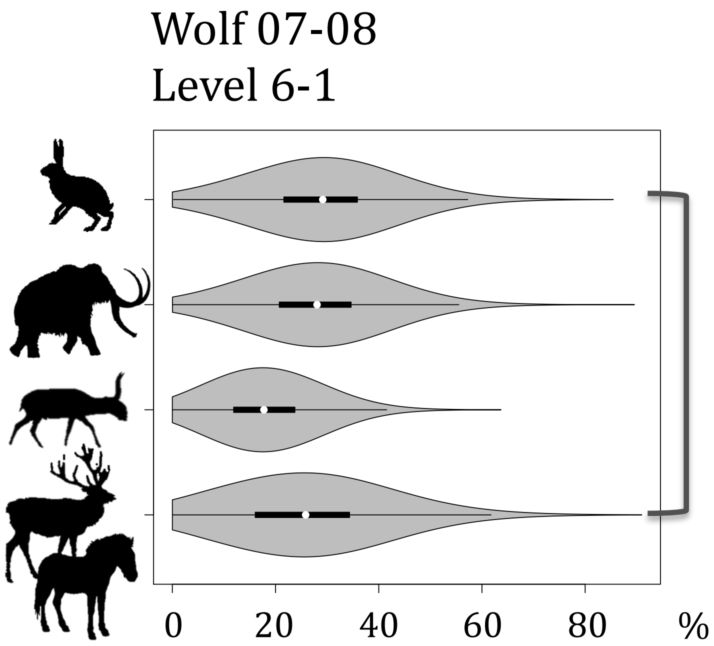

Supplementary Figure 6. Proportional contribution of Deer&Horse (red deer and horse), Saiga (saiga antelope), Mammoth (woolly mammoth) and Hare (hare) (from bottom to top along the y axis) as estimated by SIAR using the combined $\delta^{13}C_{coll}$ and $\delta^{15}N_{coll}$ values. Black boxes and whiskers show the median with 1st and 3rd quartiles and ranges with 1.5 times length of the interquartile range above the 3rd quartile or below the 1st quartile, respectively. The shaded area indicates the Kernel density plot of the probability density of prey proportions. The brackets link the resources with a significant negative correlation in their posterior distribution (animal drawings by D. Drucker).

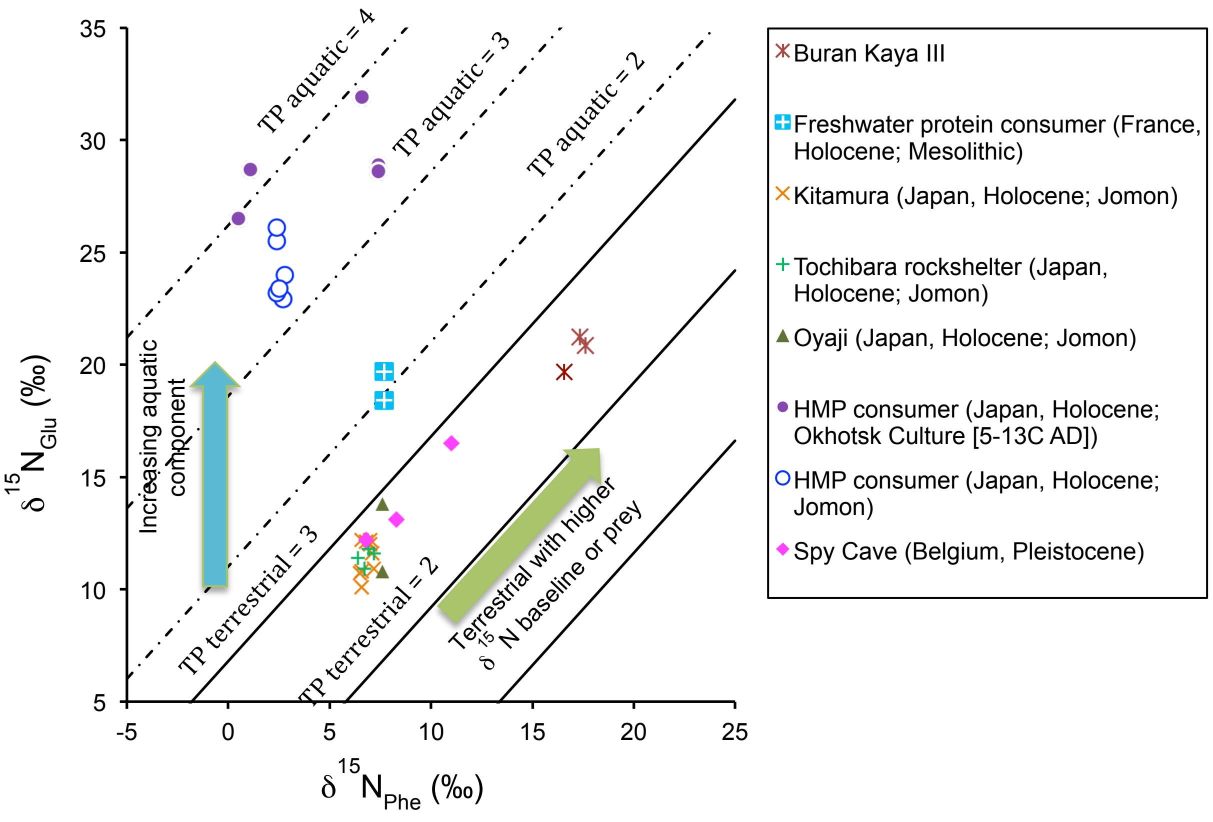

Supplementary Figure 7. $\delta^{15}N_{Phe}$ and $\delta^{15}N_{Glu}$ values of the human of Buran-Kaya III compared to a review of $\delta^{15}N_{Phe}$ and $\delta^{15}N_{Glu}$ values of archaeological humans from groups of with well-characterized diets[19]. HMP stands for High Marine Protein.

Supplementary Data 8

Isotope analyses of individual amino acids

Instrumental analysis was performed according to previous methods, with a few modifications of the equipment settings[20]. The amino acid derivatives were injected into the GC column using the Gerstel programmable temperature vaporising (PTV) injector (Gerstel, Mülheim an der Ruhr, Germany) in solvent vent mode. The PTV temperature program was as follows: 50°C (initial temperature) for 0.2 min, heating from 50°C to 250°C at the rate of 600°C min^{-1}; isothermal hold at 250°C for 10 min, heating from 250°C to 350°C at the rate of 600°C min^{-1}; and isothermal hold at 350°C for 10 min. Combustion and reduction furnaces were set at 950°C and 550°C, respectively. The GC was equipped with an Ultra-2 capillary column (50 m × 0.32 mm-i.d. 0.52-µm film

thickness; Agilent Technology). The GC oven temperature was programmed as follows: isothermal hold at 40°C for 3 min; temperature ramp to 110°C at the rate of 15°C min^{-1}; ramp to 150°C at the rate of 3°C min^{-1}; ramp to 220°C at the rate of 6°C min^{-1}; and subsequent holding isothermally at 220°C for 13 min. Carrier gas (He) flow rate through the GC column was 1.4 ml min^{-1}. CO_2 generated in the combustion furnace was eliminated by a liquid nitrogen trap. Standard mixtures of nine amino acids with known $\delta^{15}N$ values were injected into the GC/C/IRMS every five runs to confirm the reproducibility of the isotope measurements. The mean accuracy and precision of the reference mixtures were 0.0‰ and 0.4–0.7‰ (mean of 1σ), respectively. Nitrogen isotopic composition of the following 10 amino acids were determined: alanine (Ala), glycine (Gly), valine (Val), leucine (Leu), isoleucine (Ile), proline (Pro), serine (Ser), glutamic acid (Glu), phenylalanine (Phe) and hydroxyproline (Hyp). All reported $\delta^{15}N$ values for glutamic acid include a contribution from the α-amino group of glutamine, as glutamine is converted to glutamic acid during acid hydrolysis.

Supplementary Table 5. Results of the $\delta^{15}N$ values of the analysed amino acids.

Lab code	Species	$\delta^{15}N$_AA											TP
		Ala	Gly	Val	Leu	Ile	Pro	Asp+ Thr	Ser	Glu	Phe	Hyp	
BK3-07-1	*Homo sapiens*	11.8	11.9	22.7	16.8	16.3	24.6	17.7		21.2	17.4	19.7	2.6
BK3-12-1	*Homo sapiens*	12.3	15.0	20.8	16.8	19.1	25.4	16.1		20.9	17.6	16.8	2.5
BK3-07-3	*Equus* sp.	4.0	6.6	13.7	10.4	12.1	16.6	8.3	5.5	11.8	13.1	15.4	1.9
BK3-07-4	*Cervus elaphus*	9.7	4.7	10.0	4.2	0.3	13.6	6.2		10.6	13.3	7.8	1.8
BK3-07-6	*Cervus elaphus*	12.6	7.6	14.1	12.0	7.9	17.4	9.7	9.7	11.6	12.2	16.1	2.0
BK3-10-18	*Saiga tatarica*	12.6	8.3	16.0	10.8	9.1	15.9	11.9		12.4	13.3	12.3	2.0
BK3-08-04	*Saiga tatarica*	14.0	9.8	9.9	12.0	12.7	16.6	10.6		15.5	16.3	12.9	2.0
BK3-07-02	*Saiga tatarica*	11.6	6.3	8.4	10.2	6.3	14.6	8.6	5.9	12.9	14.5	14.5	1.9
BK3-07-05	*Saiga tatarica*	12.1	8.0	11.8	14.6	14.7	16.0	12.6		13.1	13.9	12.7	2.0
BK3-08-01	*Lepus* sp.	8.8	3.5	8.5	4.8	4.7	13.3	5.0		9.9	12.5	6.6	1.8

BK3-11-02	*Mammuthus primigenius*	10.2	10.3	14.7	9.6	10.1	19.1	12.1		15.5	17.5	13.7	1.8
BK3-07-08	*Canis lupus*	15.8	10.8	26.0	19.9	20.5	24.4	17.9		24.7	17.4	21.0	3.1
BK3-08-02	cf. *Vulpes vulpes*	16.2	9.2	22.3	14.1	17.1	19.2	12.9		15.8	12.3	16.4	2.6
BK3-08-03	*Vulpes* sp./*Alopex lagopus*	17.3	10.4	25.7	16.2	19.6	21.7	15.8		17.7	13.5	19.6	2.6
BK3-07-07	*Vulpes* sp./*Alopex lagopus*	14.8	6.7	18.1	15.4	17.2	21.5	12.6		18.9	14.2	17.0	2.7
BK3-11-01	*Homo sapiens*	18.3	12.8	21.9	15.5	15.1	25.4	16.4		19.7	16.6	18.0	2.5
BK3-10-19	*Saiga tatarica*	13.8	9.5	17.4	14.4	16.5	17.7	11.9		14.0	16.1	14.7	1.8
BK3-08-07	cf. *Alopex lagopus*	19.3	10.7	23.5	17.7	18.9	21.6	15.3		20.3	15.7	19.9	2.7
BK3-08-09	cf. *Alopex lagopus*	13.2	5.2	16.9	10.0	9.9	12.8	5.9	5.1	15.7	11.7	11.2	2.6

The isotopic measurement of collagen amino acids has been performed by GC/C/IRMS after optimization of instrumental settings (temperature of the reaction furnaces, carrier gas flow rate, and sample amount)[21]. Isotopic difference between proline and hydroxyproline, it might relate to possible different factors such as site taphonomy or animal phylogeny. Nevertheless, these two amino acids are not involved in the TP calculation.

Supplementary references

1. Péan, S. et al. The middle to upper Paleolithic sequence of Buran-Kaya III (Crimea, Ukraine): new stratigraphic, paleoenvironmental and chronological results. *Radiocarbon* **55**(2-3), 1454-1469 (2013).
2. Crépin, L., Péan, S. & Lázničková-Galetová, M. Comportements de subsistance au Paléolithique supérieur en Crimée: analyse archéozoologique des couches 6-2, 6-1 et 5-2 de Buran-Kaya III. *L'Anthropologie* **118**(5), 584-598 (2014).
3. Poplin, F. Essai d'ostéologie quantitative sur l'estimation du nombre d'individus. *Kölner Jahrbuch für Ur- und Frühgeschichte* **16**, 153-164 (1983).
4. Prat, S. Stress physiologique et état de santé des plus anciens Hommes anatomiquement modernes du sud-est de l'Europe (données dentaires, couche 6-1, Buran-Kaya III, Crimée). *L'anthropologie* **118**, 567-583 (2014).
5. Prat, S., et al. The Oldest Anatomically Modern Humans from Far Southeast Europe: Direct Dating, Culture and Behavior. PloS ONE **e20834**, 1-13 (2011).
6. Villa, P, et al. Cannibalism in the Neolithic. *Science* **233**, 431-437 (1986).
7. Crépin, L., Prat, S., Péan, S. & Yanevich, A. Traitement du cadavre des plus anciens hommes anatomiquement modernes de l'extrême sud-est de l'Europe (Buran-Kaya III, Ukraine). *Bull. Mém. Soc. Anthropol. Paris* **22** , 11 (2012).
8. Jürgensen, J., et al. Diet and habitat of the saiga antelope during the late Quaternary using stable carbon and nitrogen isotope ratios. *Quat. Sci. Rev.* **160**, 150-161 (2017).
9. Bekenov, A. B., Grachev, I. A. & Milner-Gulland, E. J. The ecology and management of the saiga antelope in Kazakhstan. *Mammal Rev.* **28**(1), 1-52 (1998).
10. Drucker, D. G., Bridault, A., Hobson, K. A., Szuma, E., & Bocherens, H. Can carbon-13 in large herbivores reflect the canopy effect in temperate and boreal ecosystems? Evidence from modern and ancient ungulates. *Palaeo., Palaeo., Palaeo.* **266**(1), 69-82 (2008).

11. Schmitt, J., et al. Carbon isotope constraints on the deglacial CO_2 rise from ice cores. *Science* **336**(6082), 711-714 (2012).

12. Long, E. S., Sweitzer, R. A., Diefenbach, D. R. & Ben-David, M. Controlling for anthropogenically induced atmospheric variation in stable carbon isotope studies. *Oecologia* **146**(1), 148-156 (2005).

13. Chikaraishi, Y., Ogawa, N. O. & Ohkouchi, N. Further evaluation of the trophic level estimation based on nitrogen isotopic composition of amino acids in *Earth, life, and isotopes* (ed. Ohkouchi, N., Tayasu, I., Koba, K.) 37-51 (Kyoto University Press, 2010).

14. Chikaraishi, Y., Ogawa, N. O., Doi H. & Ohkouchi N. $^{15}N/^{14}N$ Ratios of Amino Acids as a Tool for Studying Terrestrial Food Webs: A Case Study of Terrestrial Insects (Bees, Wasps, and Hornets). *Ecol. Res.* **26**(4), 835-844 (2011).

15. Richards M. P., Grimes, V. H. & Blockley, S. M. 5.4 Stable isotope analysis (C, N, O) of faunal samples in Neanderthals Among Mammoths (eds. Boismier, W.A., Gamble, C., Coward, F.) 216-219 (English Heritage, 2012).

16. Jacobi, R. M., Higham, T. F. G., Haesaerts, P., Jadin, I. & Basell, L.S. Radiocarbon chronology for the Early Gravettian of northern Europe: new AMS determinations for Maisières-Canal, Belgium. *Antiquity* **84**, 26-40 (2010).

17. Drucker, D. Validation méthodologique de l'analyse isotopique d'ossements fossiles et apports aux reconstitutions paléoécologiques du Paléolithique supérieur du sud-ouest de la France. Ph.D. Dissertation, Université de Paris 6 (2001).

18. Bocherens, H., et al. Isotopic evidence for dietary ecology of cave lion (*Panthera spelaea*) in North-Western Europe: prey choice, competition and implications for extinction. *Quat. Int.* **245**(2), 249-261 (2011).

19. Naito, Y.I . et al. An overview of methods used for the detection of aquatic resource consumption by humans: Compound-specific delta N-15 analysis of amino acids in archaeological materials. *J. Archaeol. Sci.: Reports* **6**, 720-732 (2016).

20. Chikaraishi, Y., Kashiyama, Y., Ogawa, N. O., Kitazato, H. & Ohkouchi, N. Metabolic control of nitrogen isotope composition of amino acids in macroalgae and gastropods: implications for aquatic food web studies. *Mar. Ecol. Prog. Ser.* **342**, 85-90 (2007).

21. Chikaraishi, Y., Takano, Y., Ogawa, N. O. & Ohkouchi, N. Instrumental optimization for compound-specific nitrogen isotope analysis of amino acids by gas chromatography/combustion/isotope ratio mass spectrometry. *Earth, Life, and Isotopes* **300**, 365-386 (2010).
